# Postcranial elements of small mammals as indicators of locomotion and habitat

**DOI:** 10.7717/peerj.9634

**Published:** 2020-09-02

**Authors:** Christine M. Janis, Alberto Martín-Serra

**Affiliations:** 1 School of Earth Sciences, University of Bristol, Bristol, Avon, UK; 2 Department of Ecology and Evolutionary Biology, Brown University, Providence, RI, USA; 3 Departamento de Ecología y Geología, Universidad de Málaga, Málaga, Spain

**Keywords:** Functional morpohology, Mammals, Locomotion, Habitat preference, Small body size

## Abstract

Many studies have shown a correlation between postcranial anatomy and locomotor behavior in mammals, but the postcrania of small mammals (<5 kg) is often considered to be uninformative of their mode of locomotion due to their more generalized overall anatomy. Such small body size was true of all mammals during the Mesozoic. Anatomical correlates of locomotor behavior are easier to determine in larger mammals, but useful information can be obtained from the smaller ones. Limb bone proportions (e.g., brachial index) can be useful locomotor indicators; but complete skeletons, or even complete long bones, are rare for Mesozoic mammals, although isolated articular surfaces are often preserved. Here we examine the correlation of the morphology of long bone joint anatomy (specifically articular surfaces) and locomotor behavior in extant small mammals and demonstrate that such anatomy may be useful for determining the locomotor mode of Mesozoic mammals, at least for the therian mammals.

## Introduction

The correlation of postcranial morphology with locomotor function in mammals is well known, as reviewed by Polly (2007). The median body mass of extant mammals is less than a kilogram (Blackburn & Gaston, 1998): how well does the postcranial anatomy of small mammals (i.e., <5 kg) reflect their locomotor behavior? Most functional anatomy studies of postcranial morphology in association with locomotion have focused on larger mammals, especially on primates, carnivorans, and rodents, and of course there are obvious differences between fully aquatic mammals and terrestrial ones (our focus here is on terrestrial mammals). Jenkins (1974) proposed that there was little difference between adaptations for terrestrial (i.e., ground-dwelling) vs arboreal (i.e., tree-dwelling) locomotion in small mammals: at small body sizes the perceived environment, in terms of the obstacles encountered, would be similar whether in the trees or on the ground. However Sargis (2002a) challenged that proposal, noting various authors who had reported anatomical differences between small mammals of different habitat preferences (Gebo & Sargis, 1994; Sargis, 2002a, 2002b; Argot, 2001, 2002; Szalay & Sargis, 2001): see later discussion of the work of Chen & Wilson (2015).

Mammalian species have traditionally been assigned to one of several distinct locomotor modes relating to substrate use (e.g., arboreal, scansorial (semi-arboreal), terrestrial, fossorial, semi-fossorial, semi-aquatic: see Van Valkenburgh (1987), Polly (2007), and Samuels & Van Valkenburgh (2008)). The arboreal and terrestrial modes, especially, can be further subdivided into more specialized types of locomotion: arboreal mammals can be above-branch quadrupedal climbers, below-branch suspensory climbers (usually larger mammals), vertical clingers and leapers, or gliders (the latter two categories mainly containing small mammals); terrestrial mammals can be generalized, ricochetal (hopping), ambulatory, or cursorial (the latter two categories mainly containing larger mammals). Fossorial (digging) and semi-fossorial mammals are of course terrestrial by default: these categories represent behavior rather than locomotor mode, but the functional demands of digging especially are reflected in the postcranial osteology (Hedrick et al., 2020). Although any given species may exhibit some diversity of locomotion behavior among different locomotor modes or within a particular locomotor mode (e.g., diversity of climbing behaviors in arboreal primates; see Granatosky (2018)), nevertheless there is a concept of a “locomotor mode morphotype” that typifies the habitual behavior of the species (Szalay & Dagosto, 1980), and that we apply here.

There have been numerous studies of mammalian postcrania in a comparative ecomorphological context, relating morphology to habitat preference and locomotor mode. Such studies are usually at the ordinal level (although sometimes at the level of the family), although few studies have specifically focused on small mammals. Earlier studies were primarily descriptive in nature, but over the past couple of decades later studies have demonstrated statistical correlations of mammalian limb morphology with locomotor mode, employing linear measurements and geometric morphometrics (both two dimensional and three dimensional). Dunn (2018) provides an excellent review of these methodologies and past studies on the functional anatomy of mammalian postcrania, including an evaluation of why 3-D geometric morphometrics may not always be superior to 2-D studies (see also Gould, 2014; Santana et al., 2019).

The most common taxa of interest have been primates (e.g., Szalay & Dagosto, 1980; Rose, 1988, 1989, 1994; Gebo & Sargis, 1994; Godfrey et al., 1997; Schmitt, 2003; Youlatos, 2003; Dunn et al., 2016; Elton, 2002; Elton et al., 2016; Arias-Martorell, 2018—this is but a small sample of the voluminous primate literature); carnivorous mammals (Taylor, 1974, 1976; Van Valkenburgh, 1987; Gebo & Rose, 1993; Iwaniuk, Pellis & Whishaw, 1999; Schutz & Guralnick, 2007; Polly & MacLeod, 2008; Meachen-Samuels & Van Valkenburgh, 2009; Walmsley et al., 2012; Fabre et al., 2013; Meloro et al., 2013; Janis & Figueirido, 2014; Martín-Serra, Figueirido & Palmqvist, 2014a, 2014b, 2016; Fabre et al., 2015a, 2015b; Ercoli & Youlatos, 2016; Panciroli et al., 2017; Dunn et al., 2019; Meloro & De Olivera, 2019; Tarquini, 2019); and rodents (Elissamburu & Vizcaíno, 2004; Weisbecker & Schmid, 2006; Dunn & Rasmussen, 2007; Samuels & Van Valkenburgh, 2008; Morgan, 2009; Morgan & Álvarez, 2013; Boivin et al., 2018; Calede, Samuels & Chen, 2019; Hedrick et al., 2020). Studies of other mammalian orders (excluding here larger mammals such as ungulates and large diprotodontid marsupials) include smaller marsupials (Lemelin, 1999; Argot, 2001, 2002; Szalay & Sargis, 2001; Weisbecker & Warton, 2006; Bassavora, Janis & Archer, 2009; Flores & Díaz, 2009; Warburton et al., 2011; Den Boer, Campione & Kear, 2019); scandentians (Sargis, 2002a, 2002b); lagomorphs (Reese, Lanier & Sargis, 2013); and xenarthrans (Toledo, Bargo & Vizcaíno, 2013; Amson & Nyakatura, 2018; De Oliveira & Santos, 2018). A few such comparative studies have included a diversity of mammalian orders within each study (MacLeod & Rose, 1993; Shockey, Croft & Anaya, 2007; Salton & Sargis, 2008; Croft & Anderson, 2008; Seckel & Janis, 2008; Kelly & Sears, 2011; Ercoli, Prevosti & Álvarez, 2012; Álvarez, Ercoli & Prevosti, 2013; Gould, 2014; Chen & Wilson, 2015; Figueirido, Martín-Serra & Janis, 2016: Marchi et al., 2016; DeBay & Wilson, 2017; Muñoz et al., 2017).

A seminal study of morphological correlations with locomotion in small mammals was that of Chen & Wilson (2015), showing that osteological indices (derived from linear measurements) of the long bones of small mammals could indeed distinguish different locomotor modes. Using Canonical Variates Analysis, they demonstrated that the more highly specialized types of locomotion (gliding, fossorial and ricochetal) were easily distinguished. While there was less obvious separation among more generalist mammals, there was indeed a clear “morphofunctional continuum” that could be understood in biomechanical terms. Further analyses with a more restricted set of modes of locomotion showed clearer separation among the more generalist locomotor types (i.e., arboreal, terrestrial, and scansorial).

Like Chen & Wilson (2015), our interest in the functional anatomy of the postcrania of small mammals relates to the interpretation of the locomotor behavior and habitat preferences of Mesozoic mammals, almost all of which were under five kg in size, most being of considerably smaller size than this. Chen & Wilson (2015) used their comparative dataset of extant mammals to determine the likely paleobiology of various extinct taxa that were known from at least near-complete skeletal material. However, such complete material is rare: even complete long bones are rarely preserved. The majority of postcranial remains of Mesozoic mammals consists of the dense bone at the articular ends (epiphyses) of long bones, or of small dense bones such as tarsals, and is usually not attributable to taxon (although it may be possible to distinguish metatherians from eutherians, see later discussion).

In this article we investigate the correlation of the epiphyseal anatomy of a large diversity of extant small mammals with their locomotor mode, using statistical analysis of data from 2-D geometric morphometrics. Our ultimate interest here is whether such remains of Mesozoic mammals may be used to determine the diversity of locomotor types in fossil deposits, and hence be informative about habitat structure: for example, a predominance of arboreal mammals would indicate a forested habitat, and a predominance of terrestrial ones a more open habitat type. However, application of these correlations to Mesozoic mammals will be the subject of a later article: this article is intended to serve as a review of the correlates of epiphyseal anatomy with locomotion in general, as well as a preliminary for further investigations. We followed the taxon choices of Chen & Wilson (2015), with some of our own additions, but focused only on more the generalized modes of locomotion: arboreal, scansorial, and terrestrial (including semi-fossorial). Further details of the taxa selected, and the analyses performed, are in the “Materials and Methods” section.

## Anatomy of the Articular Ends (epiphyses) of Mammalian Limb Bones

In this section we review the existing knowledge about the anatomy of mammalian limb bone joints in relation to locomotion and habitat preference, focusing on small mammals. We consider in detail only those joints that we analyzed: those which were likely to be preserved in extinct taxa, and which also yielded significant results in our analyses. This includes the shoulder, elbow, hip, and knee joints. We did not analyze the morphology of the scapula glenoid or the pelvic acetabulum, largely because of the paucity of such elements in the Mesozoic mammal fossil record. We had had high hopes for the bones of the ankle joint being a good indicator of locomotor behavior, especially as proximal tarsal elements are frequently preserved in Mesozoic fossil mammals; however, we obtained poor results for the astragalus and calcaneum (see discussion in the “Results” section), and so do not discuss them here.

### The shoulder (scapulohumeral) joint

The shoulder of therian mammals is very different from the basal mammalian condition (Jenkins & Weijs, 1979). The coracoid is lost (at least in the postnatal condition), apart from the coracoid process that is fused with the scapula and contributes to the glenoid. The therian scapula differs from the basal mammalian condition, and the monotreme condition, in the following attributes: the scapula is mobile around the dorsal border, and its movements during locomotion add to the length of the forelimb stride (Fischer et al., 2002); a supraspinous fossa (the origin for the supraspinatus muscle) and a scapular spine are new elements; and the glenoid is shallow and faces ventrally (as opposed to deep and oriented posterolaterally, or laterally in monotremes). The multituberculate shoulder approaches the therian condition: the scapula was likely mobile to some extent and the glenoid faced ventrally, although the supraspinatus fossa was small, the coracoid was retained, and the posture was more sprawling than in therians (Sereno, 2006).

During propulsion the therian forelimb basically acts as a strut around the mobile scapula, with the muscles crossing the shoulder and elbow joints acting primarily as stabilizers. This is especially the case for the shoulder joint, where the shallow glenoid does not afford a great deal of stability for the humeral head, and joint stability is maintained by the muscles of the rotator cuff that run from the scapula to the proximal humeral tuberosities (Jenkins & Weijs, 1979).

The shape of the scapular glenoid (oval, or pear-shaped) does not appear to be diagnostically different between arboreal and terrestrial forms among small mammals (Szalay & Sargis, 2001; Salton & Sargis, 2008). However, in arboreal didelphids the anterior portion is more ventrally oriented, forming a bony stop for motions of the humerus, and in terrestrial forms the glenoid cavity is hemispherical rather than elongated (Argot, 2001). In terrestrial carnivorans the glenoid is deeper than in arboreal ones (Taylor, 1974; Heinrich & Rose, 1997), and in the terrestrial (cursorial) Patas monkey (*Erythrocebus patas*) the glenoid is more square in shape (Gebo & Sargis, 1994).

The size of the humeral tuberosities (= tubercles), and whether or not they project above the humeral head, reflects the degree of stabilization of the scapulohumeral joint by the rotator cuff muscles. These comprise the supraspinatus and infraspinatus, originating from the lateral side of the scapula (from the supra- and infraspinatus fossae, respectively) and inserting onto the greater tuberosity (lateral side of the proximal humeral head); and the subscapularis, originating from the subscapularis fossa on the medial side of the scapula, and inserting onto the lesser tuberosity (medial side of the proximal humeral head) (see Janis et al. (2020), for review).

Arboreal mammals have a shoulder joint that maximizes rotational ability at the expense of stability, whereas the opposite is true in terrestrial mammals. Accordingly, arboreal mammals have a rounder, more globular humeral head, allowing for multiaxial rotation of the humerus, whereas terrestrial mammals have a more ovoid, flatter humeral head, restricting humeral motion to the parasagittal direction. Arboreal mammals have relatively small tuberosities that do not project above the level of the humeral head, reflecting not only less stabilization of the humerus on the scapula but also allowing for a greater degree of movement; terrestrial forms have larger, projecting tuberosities, especially the greater tuberosity, reflecting a greater degree of restriction of movement as well as stabilization, as large tuberosities may obstruct humeral rotation (Taylor, 1974; Heinrich & Rose, 1997; Szalay & Dagosto, 1980; Rose, 1989; Argot, 2001; Szalay & Sargis, 2001; Sargis, 2002a; Salton & Sargis, 2008; Walmsley et al., 2012; Morgan & Álvarez, 2013; Tarquini et al., 2017; Janis et al., 2020). The elongation of the humeral head is especially pronounced in diggers, which also have an extremely enlarged greater tuberosity (Salton & Sargis, 2008). Some arboreal forms may have a lesser tuberosity that is robust, and medially protruding (Sargis, 2002a; Janis et al., 2020); such morphology reflects a large subscapularis muscle, which is important for medial (internal) rotation of the humerus, and so may be important for climbing (Arias-Martorell, 2018).

In terrestrial forms the head of the humerus appears to be more “beaked”: not only deeper proximodistally, but more sharply defined at the posteroventral lip of the articular surface (Taylor, 1974; Szalay & Sargis, 2001). Taylor (1974) attributes the lack of beaking in arboreal carnivores to the presence of the insertion of a large medial triceps, which would be important in supporting the body in making a controlled descent head-first down a tree. Arboreal forms also have a more distinct bicipital groove (anteriorly, between the two tuberosities), reflecting a large tendon for the biceps brachii, a muscle important in climbers for pulling themselves up a tree, elevating the body on the forelimbs (Taylor, 1974; Heinrich & Rose, 1997; Salton & Sargis, 2008).

**Figure 1:**
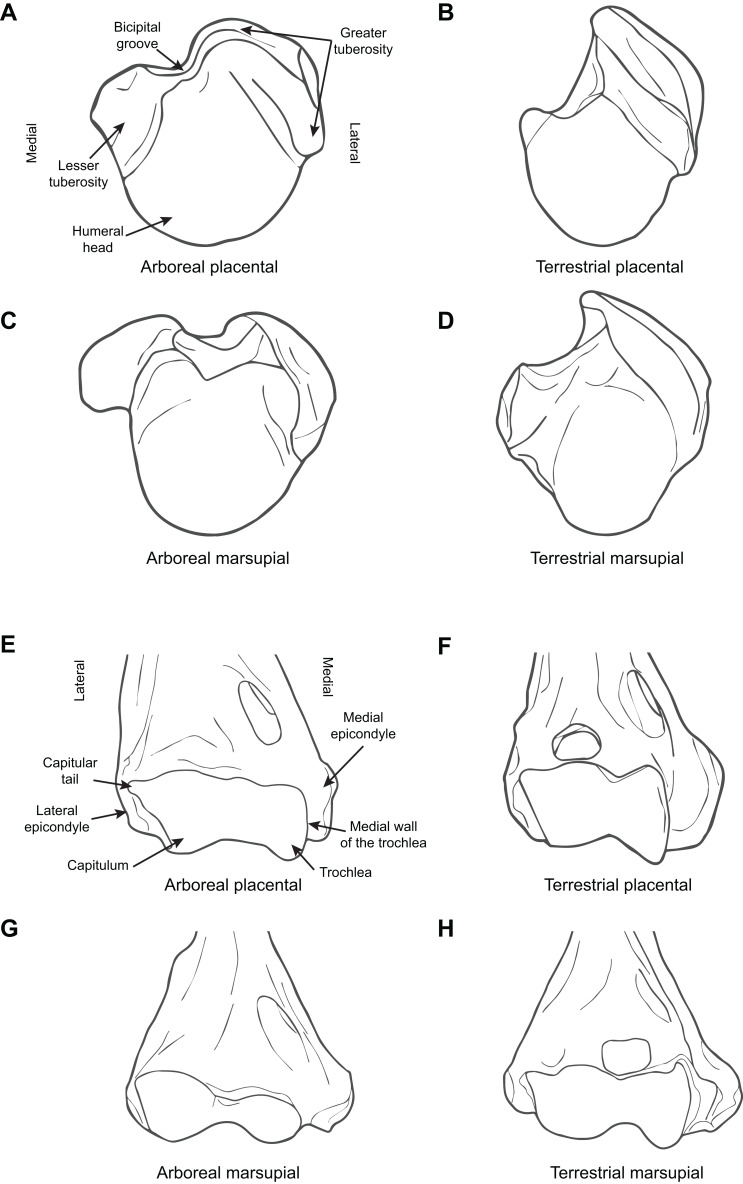
Diagrammatic depictions of humeral morphology. (A) Proximal humerus superior view, arboreal placental (*Saimiri sciurus*, based on MCZ 4247). (B) Proximal humerus superior view, terrestrial placental (*Eupleres goudotii*, based on MCZ 45958). (C) Proximal humerus superior view, arboreal marsupial (*Pseudochirus peregrinus*, based on UCMP 84683). (D) Proximal humerus superior view, terrestrial marsupial *Perameles nasuta*, based on AMNH 65659. (E) Distal humerus anterior view, arboreal placental (*Saimiri sciurus*, based on MCZ 4247). (F) Distal humerus anterior view, terrestrial placental (*E. goudotii*, based on MCZ 45958). (G) Distal humerus anterior view, arboreal marsupial (*Pseudochirus peregrinus*, based on UCMP 84683). (H) Distal humerus anterior view, terrestrial marsupial (*Isoodon obeslus*, based on UCMP 77305). All elements right hand side. Key to museum abbreviations: AMNH, American Museum of Natural History; FMNH, Field Museum of Natural History (Chicago); MCZ, Museum of Comparative Zoology (Harvard University); ROM, Royal Ontario Museum (Toronto); UCMP, University of California Museum of Paleontology (Berkeley).

Figure 1 illustrates the difference between arboreal and terrestrial taxa, showing both marsupials and placentals.

### The elbow (humeroulnar/humeroradial) joint

The elbow joint is one of the best indicators of locomotor behavior and habitat choice (substrate use) in mammals. This is because its morphology reflects not only the extent to which the body weight habitually borne on the forelimbs, and the compromise between mobility and stability (as discussed above for the humeroscapular joint), but also whether that weight is borne with the joint in a flexed or extended position. Arboreal forms moving along discontinuous, uneven support surfaces tend to have a flexed elbow while locomoting, and this also maintains their center of gravity close to the branch, important for avoiding falling. In contrast, the extended forelimb of terrestrial forms is better for propulsion along the ground (Szalay & Sargis, 2001; Sargis, 2002a). A further important function of the elbow joint is in the rotation of the forelimb in pronation and supination, a motion important for maneuverability in arboreal mammals but less important in terrestrial ones, although terrestrial mammals may need to retain this ability to a certain extent for food manipulation (Figueirido, Martín-Serra & Janis, 2016).

The therian humerus has a trochlear type of joint, in contrast to the condylar joint in nontherian mammals (Jenkins, 1973). The trochlear notch of the ulna has a spiral configuration that allows the ulna to extend in a parasagittal plane as the humerus moves through a propulsive stroke, with the result that despite the complex adduction and rotation of the humerus at the shoulder joint, the forearm remains parallel to the direction of motion of the animal (Jenkins, 1973). This reconfiguration of the elbow joint in therians may allow for the elbow to be held in a relatively adducted and retracted position during locomotion, in contrast to a more abducted position in other mammals, and may be correlated with the mobile scapula of therians that enables bounding locomotion with forelimb extension. The elbow joint is relatively similar in marsupials and placentals, with the exception of a couple of details: in marsupials (and in all Cretaceous therians), the zona conoidea between the trochlea and the capitulum articulates with the ulna, while in placentals this is an area of articulation with the radius; and the radial head tends to be circular in all marsupials whereas it varies shape with locomotor mode in placentals (see later discussion) (Szalay & Sargis, 2001).

Despite the complex morphology of the therian proximal ulna articular surface, which must necessarily be echoed in the articular surface of the distal humerus, the majority of comparative studies of the elbow joint focus on aspects of the humerus and ulna that reflect muscle attachment and lever arms. Perhaps the most studied aspect of the elbow joint is the proportions of the olecranon process of the ulna, which serves as the insertion point for the triceps muscle. In general, arboreal forms have a relatively short olecranon process that is curved forwards, reflecting a short lever arm for the triceps with a limb in a habitually flexed position; in contrast, terrestrial forms (and especially cursors) have a long, straight (or even backwardly-curved) olecranon process, reflecting a long moment arm for the triceps and a limb that is extended during propulsion (although in smaller terrestrial mammals the olecranon process is not always lengthened as is seen in larger cursorial forms) (Van Valkenburgh, 1987; Taylor, 1974; Hildebrand & Goslow, 2001; Argot, 2001; Sargis, 2002a; Salton & Sargis, 2008; Samuels & Van Valkenburgh, 2008; Ercoli, Prevosti & Álvarez, 2012, Martín-Serra, Figueirido & Palmqvist, 2014a). Digging mammals (fossorial and semi-fossorial) tend to have a particularly long (and straight) olecranon process, reflecting powerful retraction of the forelimb and hand (Hildebrand & Goslow, 2001; Salton & Sargis, 2008; Samuels & Van Valkenburgh, 2008; Martín-Serra, Figueirido & Palmqvist, 2014a).

Aspects of the anatomy of the distal humerus that are often included in comparative studies are the relative size of the epicondylar processes, including the extension of the lateral epicondylar region into a crest or ridge extending along the shaft of the humerus. These processes are the areas of origin for the hand flexors (medial epicondyle/entepicondyle) and extensors (lateral epicondyle/ectepicondyle), and so tend to be larger in mammals that do more manual manipulation, as is true for arboreal mammals but also for diggers (Taylor, 1974; Argot, 2001; Szalay & Sargis, 2001; Sargis, 2002a; Salton & Sargis, 2008). The importance of the hand flexors in arboreal mammals is not only because of the need for grasping, but also because the flexors support the body weight with the limb in a flexed position, while in terrestrial mammals the extensors support the body weight (Argot, 2001).

Despite the utility of the olecranon process of the ulna and the humeral epicondyles in determining locomotor mode in mammals, we note that they are often broken or absent in the fossils of Mesozoic mammals, whereas the articular surface themselves remain intact. We will thus focus on how the anatomy of the humeroulnar/humeroradial articulatory surfaces reflects locomotor mode, especially in small mammals. The following description is taken from Jenkins (1973), Taylor (1974), Argot (2001), Szalay & Sargis (2001), Sargis (2002a), and Szalay & Sargis (2001) emphasize how this articulation reflects the importance of positioning the hand and maintaining the forearm in a flexed position in arboreal forms, versus the loading of the forelimb in an extended position and enduring shock absorption (especially during rapid locomotion) in terrestrial ones. However, the form of the articulation differs in suspensorial arboreal forms where locking of the elbow joint is important (Szalay & Sargis, 2001): as only larger mammals (sloths and some anthropoid primates) practice this type of locomotion, this is not of concern here.

The distal humeral surface consists of the capitulum (lateral), which articulates with the radius, and the trochlea (medial), which articulates with the semilunar notch (= trochlear notch, or olecranon fossa) of the ulna, and with the coronoid process on the medial side of the notch. More arboreal forms have a spherical capitulum that allows for a large extent of movement of the radius on the humerus for pronation and supination; the trochlea is relatively small, and long and slender. In more terrestrial forms the capitulum is flatter and more spindle-shaped, and bordered by a lateral crest, the capitular tail (= the lateral flange of Szalay & Sargis (2001)). The trochlea in terrestrial forms is expanded both anteriorly and posteriorly at the expense of the capitulum, and becomes more wedge-shaped, with a medial keel. A sulcus may be present separating the capitulum from the capitular tail, which allows for the passage of the radial sesamoid (if present).

The two portions of the humeral articular surface are more equal in size terrestrial forms: in some forms they form a more continuous surface, but in others they may be separated by a depression or “gutter” (Sargis, 2002a). The size and shape of the trochlea in terrestrial forms reflects a greater amount of surface area for the articulation with the ulna, allowing for greater load absorption and repetitive loading during rapid locomotion. This morphology emphasizes joint stability over manouverablity, and the motion of the elbow joint becomes more restricted to the parasagittal plane.

The semilunar notch of the ulna reflects the shape of the humeral trochlea. The notch is both more narrow and deeper (i.e., more concave) in terrestrial forms than arboreal ones, while in arboreal forms the semilunar notch is especially wider distally. In terrestrial forms there is a prominent “beaked” anconeal process (= olecranon beak of Argot (2001), and ulnar proximal process of Szalay & Sargis, 2001), and the crests on either side of the process are wider, especially on the medial side. This anatomy allows for stability of the humeral trochlea through a wide range of motions, and the crests come into play at full extension, when they come into contact with the distal humerus, providing a stabilizing locking function (Szalay & Sargis, 2001). In particular, the crest on the lateral margin of the anconeal process contacts the posteriorly-projecting medial margin of the trochlea, effectively locking the joint in full extension (Heinrich & Rose, 1997).

The coronoid process (distal medial side of the notch, = ulnar distal trochlear crest) fits into the coronoid fossa on the distal humerus proximal to the articular surface when the elbow is fully flexed (Salton & Sargis, 2008). It extends further anteriorly in arboreal forms, allowing for stability of the joint in a flexed position, and in the absence of a prominent anconeal process and associated crests (Szalay & Sargis, 2001). In contrast, the coronoid process in terrestrial forms is deeper and more concave, articulating with the convex extension of the medial humeral trochlea (Argot, 2001). The radial notch, on the lateral side of the distal part of the semilunar notch, is for the articulation of the head of the radius. The notch faces more anteriorly in arboreal forms, where it is also more extensive in size. In terrestrial forms the notch is offset more laterally, and is more deeply set, and the articular surface also extends onto the lateral side of the coronoid process; both features reflect a radius that is more restricted in its rotational abilities (Taylor, 1974; Szalay & Sargis, 2001).

The head of the radius itself is round and relatively deep in arboreal forms, reflecting the spherical nature of the capitulum, and allowing for rotation of the elbow to permit pronation and supination. In contrast, in terrestrial forms (at least in placentals) the radial head tends to be more oval in shape, or even rectangular, and more shallow, reflecting restriction of this motion (MacLeod & Rose, 1993). While a rounded radial head is not essential for supination, this motion is facilitated if the curvature of the head abutting the ulnar surface is convex rather than flat.

**Figure 2:**
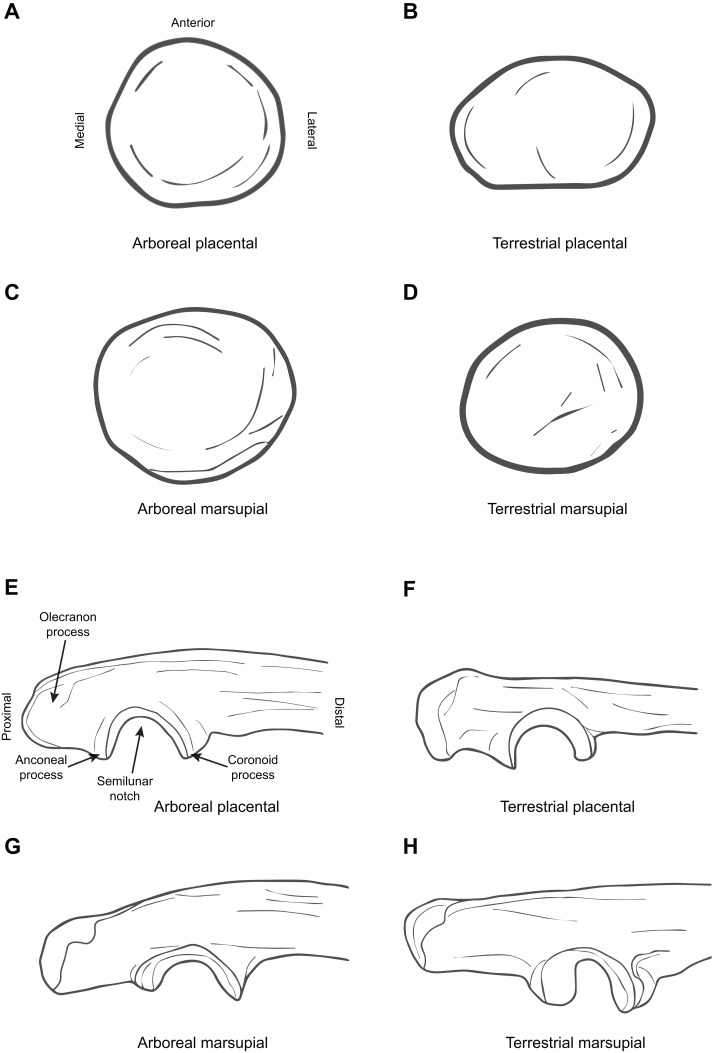
Diagrammatic depictions of ulna and radial morphology. (A) Proximal radius superior view, arboreal placental (*Loris tardigradus*, based on ROM 75742). (B) Proximal radius superior view, terrestrial placental (*Fossa fossana*, based on FMNH 85196). (C) Proximal radius superior view, arboreal marsupial (*Phalanger sericus*, based on AMNH 191203). (D) Proximal radius superior view, terrestrial marsupial (*Metachirus nudicaudatus* based on AMNH 266449). (E) Proximal ulna medial view, arboreal placental (*Callithrix jaccus*, based on MCZ 439). (F) Proximal ulna medial view, terrestrial placental (*Herpestes auropunctatus*, based on MCZ 63333). (G) Proximal ulna medial view, arboreal marsupials (*Phalanger sericus*, based on AMNH 191203). (H) Proximal ulna medial view, terrestrial marsupial (*Perameles nasuta*, based on AMNH 65659). All elements right hand side. Key to museum abbreviations as in Fig. 1.

Figure 1 illustrates the difference between arboreal and terrestrial taxa in the form of the distal humerus, showing both marsupials and placentals. Figure 2 illustrates the difference between arboreal and terrestrial taxa in the form of the proximal ulna and radius, showing both marsupials and placentals.

### The hip joint

Like the shoulder joint, the hip joint is a ball-and-socket joint, and differences between arboreal and terrestrial mammals largely reflect the ability in arboreal forms to abduct and rotate the leg, as well as the more abducted limb posture during normal stance, vs the restriction of motion in terrestrial forms to the parasagittal plane and the more adducted limb posture. These differences in turn relate to the functional demands of climbing and negotiating a variable and discontinuous substrate, vs propulsion along an even substrate always positioned directly under the body. Szalay & Sargis (2001) consider that the hind limb better reflects both locomotor mode and substrate preference than the forelimb.

Stability of the hip joint is of less concern than for the shoulder joint. There is no equivalent in the shoulder of the round ligament that affixes the head of the femur into the acetabulum in the pelvis, and thus there is no equivalent of the rotator cuff of muscles seen in the shoulder: nevertheless, analogies have been made between the stabilizing roles of the supraspinatus muscle in the shoulder and the gluteus medius muscle in the hip (Argot, 2002).

The morphology of both the acetabular articulation and femoral head reflect wide vs narrow ranges of motion, and also reflect the resting limb posture. The acetabulum faces laterally in arboreal taxa, reflecting a more abducted resting limb posture; whereas in terrestrial forms it faces ventrolaterally, and even more ventrally in diggers, reflecting hind limbs held more directly under the body (Salton & Sargis, 2009). In arboreal forms the acetabulum is elongated and elliptical in shape, relatively shallow, and with a concave dorsal border, allowing for a wide range of movements of the femoral head; while in terrestrial forms femoral motion is restricted to the parasagittal plane by a round and deep acetabulum, with a straight dorsal border (Gebo & Sargis, 1994; Szalay & Sargis, 2001; Argot, 2002; Sargis, 2002b; Salton & Sargis, 2009). The cranial expansion of the articular surface may reflect posture during climbing on vertical supports, resulting in loading from supporting the body weight on the dorso-cranial portion of the acetabulum (Sargis, 2002b).

Although the femoral head morphology broadly reflects that of the acetabulum, there are nevertheless differences between marsupials and placentals. In arboreal marsupials the femoral head is cylindrical, vs hemispherical in terrestrial forms (Szalay & Sargis, 2001; Argot, 2002); but in arboreal placentals the femoral head is only semi-cylindrical in vertical clinging and leaping primates (Anenome & Covert, 2000), and spherical and deep in most other arboreal placentals (Heinrich & Rose, 1997; Sargis, 2002b; Salton & Sargis, 2009). This difference between marsupials and placentals may be related to differences in the knee joint (see “Discussion” below), where in marsupials the asymmetry of the distal femoral condyles signifies a more extensive range of motion of the lower leg on the femur than in placentals.

In all arboreal mammals the head of the femur is relatively large, corresponding to a greater range of motion especially in abduction; the articular surface may extend onto the femoral neck (Szalay & Sargis, 2001; Sargis, 2002b; Heinrich & Rose, 1997), providing for articular surface contact when the femur is abducted (Jenkins & Camazine, 1977). Arboreal taxa have a relatively short femoral neck, while terrestrial taxa have a longer one, with the femoral head more distinct from the neck. The orientation of the head and neck to the shaft also differs, being more perpendicular in terrestrial taxa, reflecting both a less abducted resting limb posture and restriction of the limb to parasagittal motion (Jenkins & Camazine, 1977; Sargis, 2002b; Salton & Sargis, 2009). The position of the fovea capitis (the insertion of the round ligament) also differs, being located more medially and proximally in arboreal forms, reflecting the more abducted limb posture (Jenkins & Camazine, 1977; Argot, 2002).

Differences also exist between arboreal and terrestrial forms in the morphology of the femoral trochanters: the greater trochanter for the insertion of the main hip extensor and abductor, the gluteus medialis; the lesser trochanter for the insertion of the main hip flexor and adductor, the iliopsalis; and the third trochanter, for the insertion of the gluteus superficialis. Differences here relate to the demands for slower and more powerful motions of the hip joint in arboreal forms, reflecting elevation of the body as well as propulsion, versus an emphasis on speed rather than power in terrestrial forms, especially in hip flexion.

In terrestrial forms the greater trochanter is larger, projecting higher than the femoral head, providing a longer moment arm for the medial gluteus, and reflecting the more powerful extension of the hip joint for “push off” in terrestrial locomotion (Heinrich & Rose, 1997; Gebo & Sargis, 1994; Argot, 2002; Szalay & Sargis, 2001; Sargis, 2002b; Salton & Sargis, 2009). However, as with the height of the greater tuberosity in the humerus, a long greater trochanter limits the ability to abduct the limb (Sargis, 2002b). The shorter greater trochanter of arboreal forms reflects the more habitually flexed position of the hind limb (Sargis, 2002b). In contrast, the lesser trochanter is larger in arboreal forms, and it is also oriented more distally and medially, reflecting powerful (rather than rapid) limb protraction and also lateral rotation (Taylor, 1976; Heinrich & Rose, 1997; Szalay & Sargis, 2001; Argot, 2002; Sargis, 2002b; Salton & Sargis, 2009). The smaller, more posteriorly and proximally placed lesser trochanter in terrestrial forms reflects rapid hip flexion (Argot, 2002), presumably during the recovery phase of the limb cycle. The third trochanter is variably present among mammals, its absence related more to phylogeny than to function (Sargis, 2002b; Salton & Sargis, 2009). In mammalian lineages where it is present (e.g., prosimian primates, tupaiids, some carnivorans), the third trochanter tends to be larger and laterally expanded in terrestrial forms, and more distally positioned, especially in diggers (Salton & Sargis, 2009).

**Figure 3:**
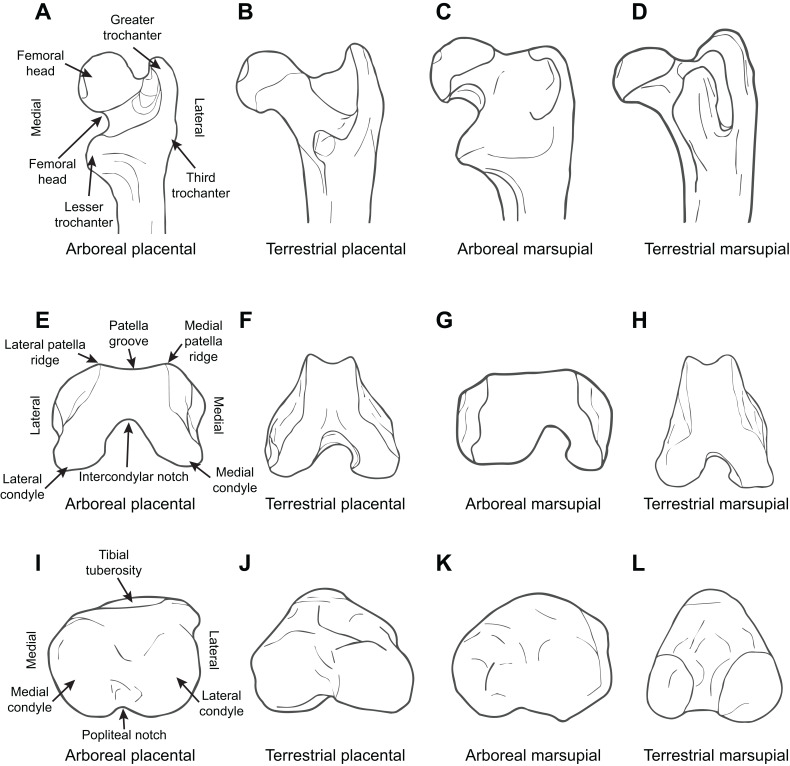
Diagrammatic depictions of femoral and tibial morphology. (A) Proximal femur posterior view, arboreal placental (*Perodicticus potto*, based on ROM 77539). (B) Proximal femur posterior view, terrestrial placental (*Fossa fossana*, based on FMNH 85196). (C) Proximal femur posterior view, arboreal marsupial (*Caluromys lanatus*, based on MCZ 37857). (D) Proximal femur posterior view, terrestrial marsupial (*Perameles bougainville*, based on MCZ 52970). (E) Distal femur inferior view, arboreal placental (*P. potto*, based on ROM 77539). (F) Distal femur inferior view, terrestrial placental (*F. fossana*, based on FMNH 85196). (G) Distal femur inferior view, arboreal marsupial (*C. lanatus*, based on MCZ 37857). (H) Distal femur inferior view, terrestrial marsupial (*P. bougainville*, based on MCZ 52970). (I) Proximal tibia superior view, arboreal placental (*P. potto*, based on ROM 77539). (J) Proximal tibia superior view, terrestrial placental (*F. fossana*, based on FMNH 85196). (K) Proximal tibia superior view, arboreal marsupial (*C. lanatus*, based on MCZ 37857). (L) Proximal tibia superior view, terrestrial marsupial (*Macrotis lagotis*, based on AMNH 35685). All elements right hand side. Key to museum abbreviations as in Fig. 1.

Figure 3 illustrates the difference between arboreal and terrestrial taxa in the form of the proximal femur, showing both marsupials and placentals.

### The knee (femorotibial) joint

As with the joints in the forelimb, a prime difference between arboreal and terrestrial taxa is the issue of mobility in the former and stability in the latter. Yet the form of the knee articulation is more related to locomotor and postural behaviors than to substrate use (Szalay & Sargis, 2001; Argot, 2002). This may be because the hind limb has a different role in locomotion from the forelimb: it is more concerned with the generation of propulsive force, and less concerned with absorbing repetitive impacts on landing (although the latter might not be as true for bipedal forms).

Unlike the other joints considered here, the knee joint shows some profound differences between marsupials and placentals. This is an especially important consideration for small mammals, as the differences are much less among more terrestrial forms in both groups, and larger mammals tend to be more terrestrial in their habits. A prime anatomical difference is in the metatherian lack of a patella: a patella appears to be a basal eutherian feature, although several clades of specialized terrestrial marsupials (caenolestids (possum rats), notoryctids (marsupial moles), and peramelids (bandicoots and bilbies)) have independently evolved a patella (Szalay & Sargis, 2001). However, the lack of a patella has surprisingly little effect on the one aspect of femoral morphology that might be expected to show a difference—the length and form of the patella groove—as will be discussed below. The real difference seen in the distal femur is in the relative sizes of the femoral condyles. The generalized condition in marsupials, retained in all but specialized terrestrial forms, is for the lateral condyle to be considerably larger than the medial one, while in placentals the condyles are more or less subequal in size in all locomotor types (Szalay & Sargis, 2001). In placentals the medial condyle may be the slightly larger one (Salton & Sargis, 2009). These differences in the femoral condyles are reflected in the tibial articulatory surfaces: the medial and lateral tibial condyles are of similar size in small placentals, but in all but highly specialized terrestrial marsupials the lateral one is larger than the medial one (Szalay & Sargis, 2001; Argot, 2002). In terrestrial therians the tibial condyles tend to be slightly different in shape, with the lateral condyle being slightly convex and the medial one concave, which increases the stability of the knee articulation (Argot, 2002).

Szalay & Sargis (2001) propose that this difference in the symmetry of the femoral condyles reflects the different ancestral habits of the earliest members of the two therian lineages, metatherians having an arboreal ancestry and eutherians a terrestrial one. This difference in morphology relates to the somewhat abducted position of the hind limb in most marsupials (e.g., *Didelphis*, see Jenkins, 1971); with the attainment of a less abducted position of the lower leg during push-off with the foot (i.e., a more parasagittal stance), reduction of the lateral condyle would bring the hind limb under the body more rapidly during locomotion (Szalay & Sargis, 2001).

In terrestrial therians, in comparison with arboreal forms, the distal femur is longer in an anterior–posterior direction (i.e., deeper in distal view), with a deeper and narrower patella groove with more prominent margins (Taylor, 1976; Heinrich & Rose, 1997; Szalay & Sargis, 2001; Argot, 2002; Sargis, 2002b; Salton & Sargis, 2009; Gould, 2014). This anatomy increases the moment arm for the tendon of the quadriceps muscle (which is enhanced by the presence of a patella), and the mechanical advantage of the muscle itself in extending the lower leg. The morphology of a deeper distal femur is mirrored by a more prominent tibial tuberosity (where the quadriceps tendon inserts), resulting in a more triangular-shaped proximal tibial articulatory surface (Heinrich & Rose, 1997; Argot, 2002; Sargis, 2002b). Among small mammals, this knee anatomy is especially prominent (along with femoral condyles that are subequal in size) in specialized cursors, both in placentals (e.g., the bounding elephant shrew, *Petrodromus tetradactylus* (Salton & Sargis, 2009)), and marsupials (bounding peramelids and the hopping kultaar, *Antechinomys laniger* (Argot, 2002)). However, a similar anatomy can also be seen in some small arboreal primates, in leapers such as galagos, that require rapid propulsion generated by the hind limb (Szalay & Sargis, 2001; Sargis, 2002b; Salton & Sargis, 2009). Some small terrestrial placentals can have more shallow and wide patella grooves, as seen in diggers such as the lipotyphlan *Solenodon paradoxurus* (the solenodon) and the tenrecoids *Oryzorictes hova* and *O. tetradactylus* (rice or mole tenrecs), probably related to the lateral abduction and rotation of the lower limb while bracing with the hind limb during digging with the forelimbs (Salton & Sargis, 2009).

While terrestrial forms usually need restricted and rapid movement at the knee, arboreal forms can benefit from having a knee that allows for a greater degree of independent rotation of tibia in relation to the femur, especially in postures where the body is oriented relative to fixed feet, as in grabbing a branch and reaching with the forelimbs (Argot, 2002). In arboreal mammals stability at the knee joint is achieved via extensive ligamentous connections rather than by the form of the articulatory surfaces (Argot, 2002). The femoral condyles are larger in terrestrial forms and project more posteriorly, increasing the leverage of the quadriceps by increasing the diameter of the pulley formed by the distal epiphysis; however, the actual articular surfaces project more posteriorly in arboreal forms, reflecting a more habitual posture with a flexed knee (Argot, 2002; see also Gould, 2014).

Figure 3 illustrates the difference between arboreal and terrestrial taxa in the form of the distal femur and proximal tibia, showing both marsupials and placentals.

## Materials and Methods

We employed 2D geometric morphometrics on the limb bone epiphyses of 76 species of extant small (<5 kg) therian mammals (24 marsupials, 52 placentals) of known locomotor mode (arboreal, scansorial, terrestrial). Usually only one individual was sampled. Although it would have been preferable to have sampled multiple individuals for each taxon, postcranial material is scarce in museum collections. Additionally, especially in the case of small mammals, the limb elements are often in articulation and bound by ligaments, and so the articulatory surfaces cannot be photographed. In the instances where more than one individual was included, this was usually to make up for missing limb elements in the original specimen. In order to avoid taxonomic over-sampling, we usually only included one species of each genus; occasionally a second species of a genus was included to enable sampling of each element. Table 1 shows an abbreviated listing of the taxa included and their locomotor affinity: a full accounting, including the taxonomic abbreviations used in the figures and the elements used for each individual animal, can be found in Tables S1–S4.

**Table 1:** Summary of taxa used in these analyses, including numbers in three different locomotor categories. A more complete listing of the taxa sampled can be found in Tables S1–S4.

Order	Family	No. of species	# Arboreal	# Scansorial	# Terrestrial
Marsupialia					
Didelphimorphia (Ameridelphia)	Caenolestidae	1	0	1	0
	Didelphidae	6	1	4	1
Dasyuromorphia (Australodelphia)	Dasyuridae	7	1	3	3
Diprotodontia (Australodelphia)	Acrobatidae	1	1	0	0
	Petauridae	3	3	0	0
	Phalangeridae	2	1	1	0
	Pseudocheiridae	1	1	0	0
Peramelemorphia (Australodelphia)	Peramelidae	3	0	0	3
Placentalia					
Afrosoricida	Tenrecidae	3	0	0	3
Eulipotyphla	Erinaceidae	1	0	0	1
	Solenodontidae	1	0	0	1
	Soricidae	1	0	0	1
Scandentia	Tupaiidae	2	0	2	0
Primates (Lemuriformes)	Cheirogaleidae	2	2	0	0
Primates (Anthropoidea)	Callitrichidae	1	1	0	0
	Cebidae	1	1	0	0
	Pithecidae	1	1	0	0
Rodentia (Caviomorpha)	Caviidae	2	0	0	2
	Chinchillidae	1	0	0	1
	Cuniculidae	1	0	0	1
	Dasyproctidae	1	0	0	1
	Echimyidae	2	2	0	0
	Erethizontidae	2	2	0	0
Rodentia (Myomorpha)	Cricetidae	3	0	1	2
	Gliriidae	1	1	0	0
Rodentia (Sciuromorpha)	Sciuridae	6	0	4	2
Carnivora (Caniformia)	Mephitidae	1	0	0	1
	Mustelidae	2	0	1	1
	Procyonidae	6	3	3	0
Carnivora (Feliformia)	Eupleridae	5	0	2	3
	Herpestidae	2	0	0	2
	Nandinidae	1	1	0	0
	Prionodontidae	1	0	1	0
	Viverridae	2	0	1	1

The mammal species were assigned to the following locomotor modes (from information in a diversity of literature sources, including that in Chen & Wilson (2015)): arboreal (primarily living in trees, rarely locomoting on the ground, 22 taxa); terrestrial (primarily living on the ground, almost never climbing trees, 30 taxa); scansorial (= semi-arboreal, regularly locomoting both on the ground and within the canopy, 24 taxa). We included semi-fossorial taxa with the terrestrial forms, and excluded the specialized fully-fossorial taxa. We also excluded terrestrial forms that had other kinds of specialized locomotion, such as ricochetal (seen in several rodents and in macropodids) and cursorial (seen in lagomorphs, macroscelideans, and canid and felid carnivorans, although we did include a few semi-cursorial rodents such as the agouti (*Dasyprocta punctata*)). We also excluded small xenarthrans, due to their highly specialized anatomy. Some other small mammals that were initially included were later excluded, as their morphology appeared highly specialized in comparison to other similar forms in the sample: these included hyraxes (Hyracoidea) and lorises and potos (Primates: Lorisidae). Hyracoids had especially unusual morphologies of the proximal humerus, and lorisids of the proximal ulna. Our intention was to create a dataset of animals of relatively homogenous morphologies for their locomotor type, and we did not want the statistical significance of any one locomotor category to be driven by the extreme morphology of essentially outlier taxa.

**Figure 4:**
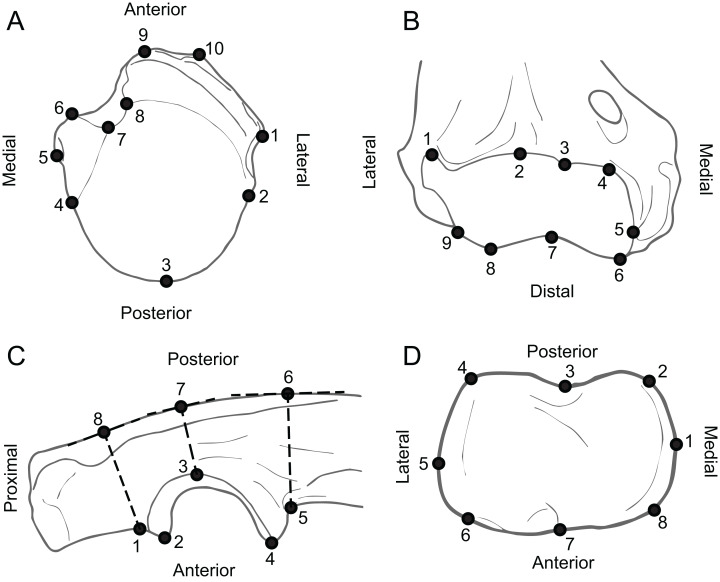
Anatomy of forelimb epiphyses, illustrating the landmarks used for the Geometric Morphometric analyses. (A) Proximal humerus (superior view, based on *Ailurus fulgens* MCZ 64643). (B) Distal humerus (anterior view, based on *Mustela nigripes* MCZ 42737). (C) Proximal ulna (medial view, based on *Nandinia binotata* AMNH 51461). (D) Proximal radius (superior view, based on *Dasyprocta punctata* MCZ 5094). All elements right hand side. Key to museum abbreviations as in Fig. 1. See the Table S5 for detailed information as to the position of the landmarks.

**Figure 5:**
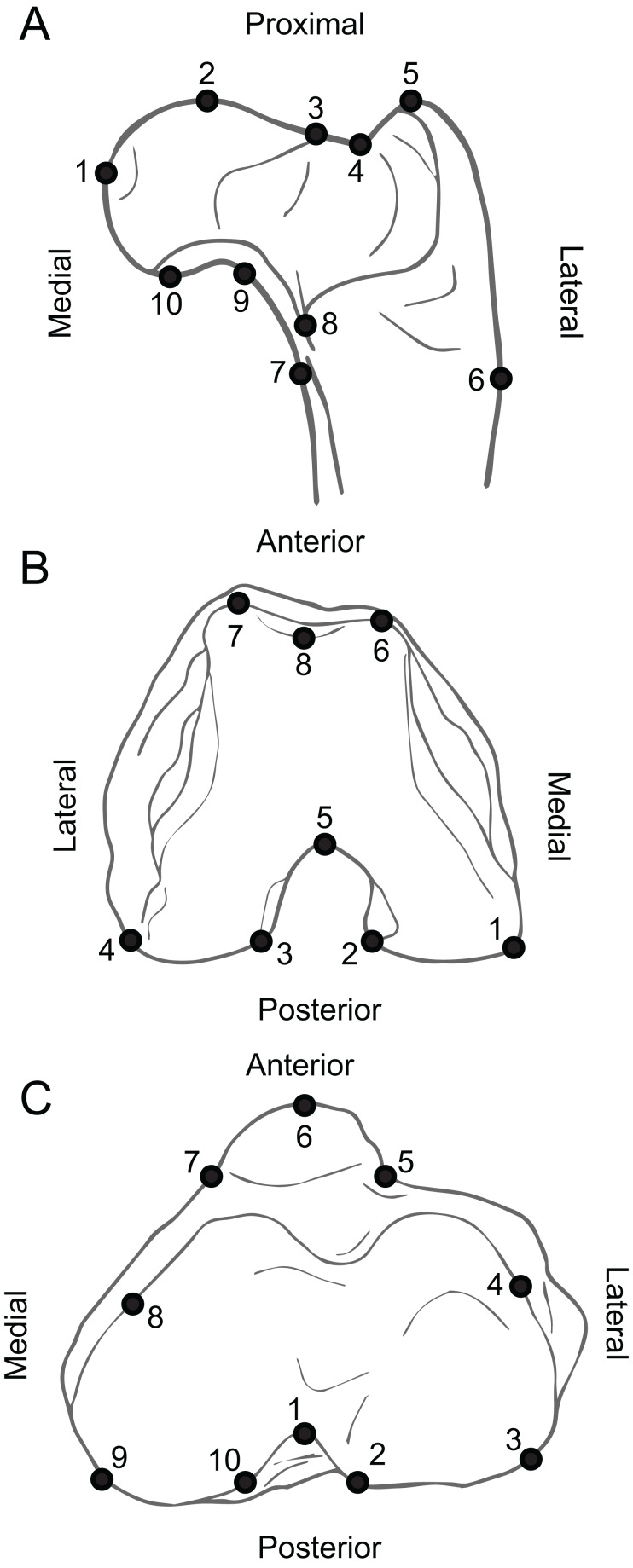
Anatomy of hind limb epiphyses, illustrating the landmarks used for the Geometric Morphometric analyses. (A) Proximal femur (posterior view, based on *Mustela nigripes* MCZ 42737). (B) Distal femur (inferior view, based on *M. nigripes* MCZ 42737). (C) Proximal tibia (superior view, based on *Ailurus fulgens* MCZ 64643). All elements right hand side. Key to museum abbreviations as in Fig. 1. See the Table S5 for detailed information as to the position of the landmarks.

Specimens were photographed in the collections of a diversity of institutions (see legend for Fig. 1). All specimens were photographed with a scale bar, either with a Nikon DSLR camera (larger ones), or with a Celestron Digital Microscope Pro connected to a MacBook Air (smaller ones). The proximal humeri were photographed in superior view (similarly to Janis et al. (2020) and Fig. 4A). The distal humeri were photographed in anterior view, such that most of the surface of the trochlea and capitulum, as well as their distal projections, were observable (Fig. 4B). The proximal ulnae were photographed in medial view, such that the depth of the semilunar notch and the projection of the anconeus and coronoid processes were visible (Fig. 4C). The proximal radii were photographed in superior view such that the shape of the proximal articular surface was defined (Fig. 4D). The proximal femora were photographed in posterior view, such that femoral head and all trochanters were visible (Fig. 5A). The distal femora were photographed in inferior view, such that the patellar groove and both condyles were observable (Fig. 5B). The proximal tibiae were photographed in superior view, such that both condyles and the tibial tuberosity were visible (Fig. 5C). Some of these elements were also photographed in other views: the proximal femur in superior view, and the proximal ulna in anterior and lateral views. None of these alternative views yielded good results in analyses.

The data were analyzed in a similar fashion as described in Janis et al. (2020). A set of eight to ten 2D landmarks were selected for each bone epiphysis following a criterion of homology and morphological relevance (Figs. 4 and 5; Table S5). We avoided high-dimensional semi-landmarks curves because the overload of dimensions is not recommended for some of the subsequent analyses (Canonical Variate Analysis (Mitteroecker & Bookstein, 2011), see below). This process was developed using the TPS Util 1.68 and TPS Dig 2.25 (Rohlf, 2016a, 2016b).

The raw landmark coordinates were imported into the software MorphoJ (Klingenberg, 2011). In order to remove the differences in size, translation and rotation, a Procrustes alignment (Dryden & Mardia, 1998) was performed for the epiphysis of each bone. To test the association between shape and locomotor groups independently of size and phylogenetic structure, a series of phylogenetic Procrustes ANOVAs were carried out using these Procrustes coordinates. To do this, we downloaded a phylogeny of therian mammals that included our sampled species from vertlife.org (Upham, Esselstyn & Jetz, 2019) (see Fig. S1), and this tree was then pruned to match with the species available for each bone epiphysis. We then incorporated the phylogeny and the Procrustes coordinates into the R environment (R Development Core Team, 2017) and performed a series of phylogenetic Procrustes ANOVAs using the function *procD.pgls* of *geomorph* package (Adams et al., 2017) with log-transformed centroid size and locomotor categories as independent variables.

The Procrustes coordinates of each bone epiphysis were used to carry out a Principal Components Analysis (PCA) and a Canonical Variate Analysis (CVA). The CVAs were performed classifying each species into one of the three locomotor categories described above. They were performed using two statistical software packages: MorphoJ (Klingenberg, 2011) to obtain the shape changes of each canonical axis and the results of the significance permutation tests for Mahalanobis and Procrustes distances; IBM SPSS Statistics v.15 was used to obtain the percentages of correct classification using leaving-one-out cross-validation method. obtained from each CVA. As a cautionary note, this method is completely valid because we have tested that CVA results obtained from both packages are identical by regressing their canonical functions (*r*^2^ = 0.9995 to 1; slope = 0.9997 to 1.0027). The values of the Procrustes coordinates for each individual and each bone are presented in Table S6.

## Results

Several limb features were well-differentiated between locomotor groups, and were able to classify taxa to their known locomotor group (arboreal, scansorial, or terrestrial) with greater than 70% accuracy (or greater than 50% with cross validation) (see Table 2), and showed significant differences at distinguishing among the different groups (see Table 3) in the canonical variates analyses. Almost all of the phylogenetic Procrustes ANOVAs performed for each bone epiphysis yielded significant results for the separation between locomotor categories, with the exception of the proximal radius and tibia (Table S6). In addition, size does not seem to be an important factor as its effect was significant only for two epiphyses (proximal ulna and proximal femur).

**Table 2:** Performance of limb elements in the canonical variates analysis 1.

Element	% Arb	%A as T	% Scans	% Terr	%T as A	TC%	TC% XV
Proximal humerus	55	22	48.1	48.4	15	71	50
Distal humerus	60	12	42.3	44.1	5	67.5	47.4
Distal humerus + medial epi.	77	6	33.3	51.9	14.8	76.9	52.3
Proximal ulna	68.4	3.2	45.8	61.3	5.3	78.4	58.1
Proximal ulna + olecranon pr.	52.6	10.5	45.5	78.1	3.1	79.5	61.6
Proximal radius	40	20	44.4	42	23.1	67.8	42.4
Proximal femur	55.6	27.8	74.1	44.8	34.5	81.1	58.1
Distal femur	65	20	67.9	51.4	10	77.1	60.2
Proximal tibia	18.2	27.3	31.3	42.9	19	77.1	33.3
Calcaneum	11.1	55.6	15.4	15.8	36.8	51.2	14.6

**Note:**

Comparison of percent classification into different locomotor groups. %Arb, percent correctly classified as arboreal; %Scans, percent correctly classified as scansorial; %Terr, percent correctly classified as terrestrial; %A as T, percent arboreal forms incorrectly classified as terrestrial; %T as A, percent terrestrial forms incorrectly classified as arboreal; TC%, total % correctly classified; TC%XV, total % correctly classified with cross validation; epi., epicondyle; pr., process. Results for the astragalus were even poorer than for the calcaneum, and are not shown here.

**Table 3:** Performance of limb elements in the canonical variates analysis 2.

Element	Mahalanobis distance			Procrustes distance		
	A vs S	A vs T	S vs T	A vs S	A vs T	S vs T
Proximal humerus	**<0.0001**	**<0.0001**	**0.038**	**0.0028**	**<0.0001**	0.2112
Distal humerus	**<0.0001**	**<0.0001**	0.4751	**0.0011**	**0.0001**	0.4593
Distal humerus + medial epi.	**<0.0001**	**<0.0001**	0.2326	**0.0355**	**0.0166**	0.3636
Proximal ulna	**<0.0001**	**<0.0001**	**0.0002**	0.057	**0.0003**	0.1324
Proximal ulna + olecranon pr.	**<0.0001**	**<0.0001**	**<0.0001**	0.1804	**<0.0001**	**0.0003**
Proximal radius	**0.0165**	**0.0057**	**0.0359**	**0.0153**	**0.0073**	0.2291
Proximal femur	**<0.0001**	**<0.0001**	**<0.0001**	**0.047**	**0.005**	**0.0161**
Distal femur	**<0.0001**	**<0.0001**	**<0.0001**	0.0608	**<0.0001**	0.0373
Proximal tibia	0.4085	**0.0001**	**0.0003**	0.0591	0.06	0.0637
Calcaneum	0.8843	0.9612	0.948	0.8722	0.9782	0.8959

**Note:**

Probabilities of pairwise comparison of locomotor groups. A vs S, arboreal vs scansorial; A vs T, arboreal vs terrestrial; S vs T, scansorial vs terrestrial; epi., epicondyle, pr., process. Values with significance levels <0.05 are in bold.

The humerus, ulna and femur all showed good discrimination: all of these elements (both proximal and distal ends in the case of the humerus and femur) could distinguish between arboreal and terrestrial taxa, and also arboreal and scansorial taxa, with a significance level of at least *p* < 0.05 (and in many cases *p* < 0.0001), although distinction between scansorial and terrestrial taxa was more elusive (especially with the Procrustes differences).

The proximal femur was the only element that could always distinguish the scansorial forms, and the distal femur (which had the best cross-validation reclassification scores) was able to distinguish scansorial forms in all instances except the Procrustes distances of arboreal vs scansorial. In contrast, the forelimb elements were almost always able to distinguish scansorial forms from arboreal ones, but were less likely to be able to distinguish them from terrestrial ones (and here the distal humerus was the poorest performer). We also analyzed (but do not show the plots of the analyses) the distal humerus with the inclusion of the medial epicondyle and the proximal ulna (medial view) with the inclusion of the olecrcanon process. Note that in both cases, the percentage of forms correctly classified was slightly better (see Table 3), but the significance values remained unchanged (except for the Procrustes distances in distinguishing between scansorial and terrestrial forms in the case of the ulna).

The proximal radius and the distal tibia did not yield as good a result as did the above-mentioned bones. In the case of the proximal radius, this may be in part because there is much less variation in shape among marsupials than among placentals (see later discussion), although this element could still correctly classify ~68% of taxa (~42% with cross validation), and for the most part could distinguish among the locomotor categories (although with lower levels of significance than for the humerus, ulna, and femur). Photographs of the proximal radius were difficult to obtain (as it was often attached to the ulna), and only 69 specimens were included in the analyses. In the case of the proximal tibia, the ability to classify taxa was poorer than that of the radius, and there were few instances where the locomotor groups could be distinguished from one another. Obtaining data on the proximal tibia was also problematical: not only was the articular surface difficult to photograph (as it was often obscured by soft tissues), but the edges of the articular surface in many of the small mammals were indistinct, making it difficult to place the geometric markers. The morphological changes picked up by the analyses appear to be subtle, at best, making it difficult to interpret any functional differences between taxa. Good images of the proximal tibia were available for only 46 species (see Tables S1–S4).

As noted in the introduction, we achieved very poor results for the astragalus (anterior view) and calcaneum (both anterior and lateral views). The lateral view was the best indicator and this is the one shown in the tables: only ~51% of the taxa were correctly classified (and only ~15% with cross validation), and none of the locomotor groups could be distinguished from each other. An additional problem was the low samples for these bones, in part because they were either lacking entirely in the specimens available to us (sometimes being preserved as part of the foot in the skins), or they were so tightly bound by ligaments to each other and/or to the tibia that it was not possible to obtain a good image. Good images of the calcaneum were available for only 41 species, and for the astragalus only 26 species (see Tables S1–S4).

Below we discuss the results for each bone in more detail (with the exception of the astragalus and calcaneum, which we elaborate on further in the “Discussion” section). A summary of the different “performance” of each element is provided in Table 4.

**Table 4:** Summary of the performance of the various limb elements.

Element	Ability to reclassify taxa	Distinguishes Arb. from Terr.?	Distinguishes Arb. from Scan.?	Distinguishes Terr. from Scan.?
Proximal humerus	71% (50%)	**Always**	**Always**	Sometimes
Distal humerus	68% (47%)	**Always**	**Always**	No
with medial epicondyle	77% (52%)	Always	Always	No
Proximal ulna	78% (58%)	**Always**	Sometimes	Sometimes
with olecranon process	80% (62%)	**Always**	Sometimes	**Always**
Proximal radius	68% (42%)	Always	Always	Sometimes
Proximal femur	80% (55%)	**Always**	Always	Always
Distal femur	77% (60%)	**Always**	Sometimes	Sometimes
Proximal tibia	77% (33%)	Sometimes	No	Sometimes
Calcaneum	51% (15%)	No	No	No

**Note:**

The reclassification percentages have been rounded up from Table 2, and the figures in parentheses show the percentages obtained by cross validation. For the ability to distinguish between pairs of locomotor types: “Always” = distinguished by both Mahalanobis and Procrustes distances, and “Sometimes” = distinguished by Mahalanobis distances only. Arb., arboreal; Scan., scansorial; Terr., terrestrial. A bolded term means that the significance level is *p* < 0.005.

### Proximal humerus

#### Principal Components Analysis

**Figure 6:**
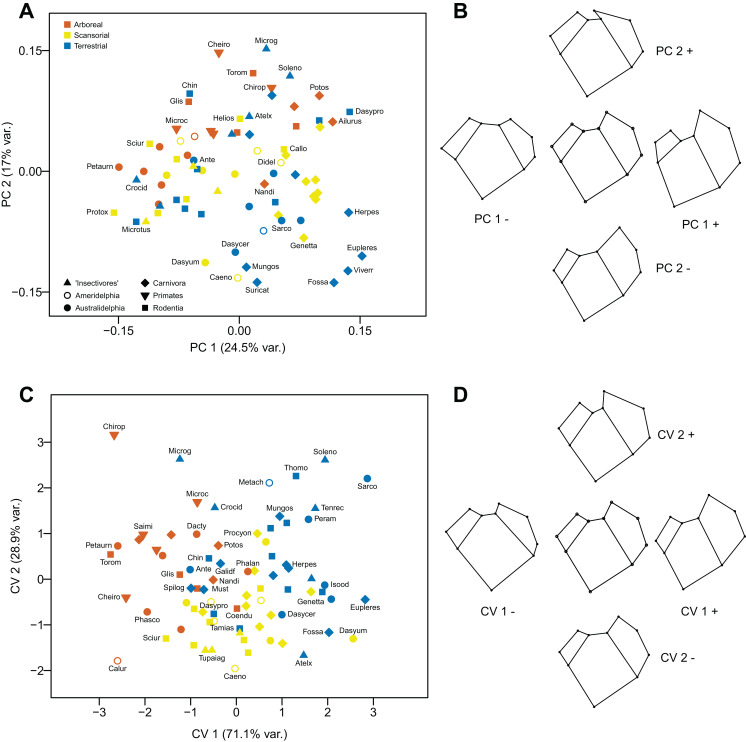
Analyses of the proximal humerus (superior view). (A) Principal components analysis. (B) Shape changes associated with each PC plus the consensus shape (center). (C) Canonical variates analysis. (D) Shape changes associated with each CV plus the consensus shape (center).

The first component (explaining 24.5% of the variance) distinguishes proximal humeri with a more ovoid humeral head, a relatively larger greater tuberosity and a relatively smaller lesser tuberosity (positive values), from humeri with a more rounded humeral head, a relatively small greater tuberosity and a relatively large lesser tuberosity (negative values). The second component (explaining 17% of the variance) distinguishes taxa primarily on the shape of the greater tuberosity: humeri with the cranial portion of the tuberosity projecting medially (and a slightly larger lesser tuberosity) have positive values, while humeri where the cranial portion of the tuberosity projects anteriorly (and a slightly smaller lesser tuberosity) have negative values. The majority of the terrestrial taxa have positive scores on the first component. Arboreal taxa tend to have positive scores on the second component and terrestrial taxa tend to have negative scores (see Figs. 6A and 6B).

#### Canonical Variates Analysis

The differences in humeral morphology along the first axis are similar to those along PC1, although there is less difference in the size of the lesser tuberosity between positive and negative values. The morphological changes along the second axis differ from those in PC2 (although the differences in the size of the lesser tuberosity are similar): here humeri with larger greater tuberosities have more positive values although, as with the second component of the PCA, the cranial portion projects medially. The first axis (explaining 71.1% of the variance) largely separates terrestrial taxa (positive scores) from arboreal ones (negative scores), with scansorial taxa lying in between these two groupings. The second axis (explaining 28.9% of the variance) does not appear to contain a strong locomotor signal. Marsupials group with the similar locomotor group of placentals (see Figs. 6C and 6D).

Using Mahalanobis distances between groups, the CVA distinguishes all three locomotor categories from each other; using Procrustes distances arboreal taxa are distinguished from scansorial and terrestrial ones with a high degree of significance, but scansorial and terrestrial taxa cannot be distinguished from one another. Out of the original grouped taxa, 70.5% were correctly classified (50% for cross-validated groups) (see Table 2).

### Distal humerus

#### Principal Components Analysis

**Figure 7:**
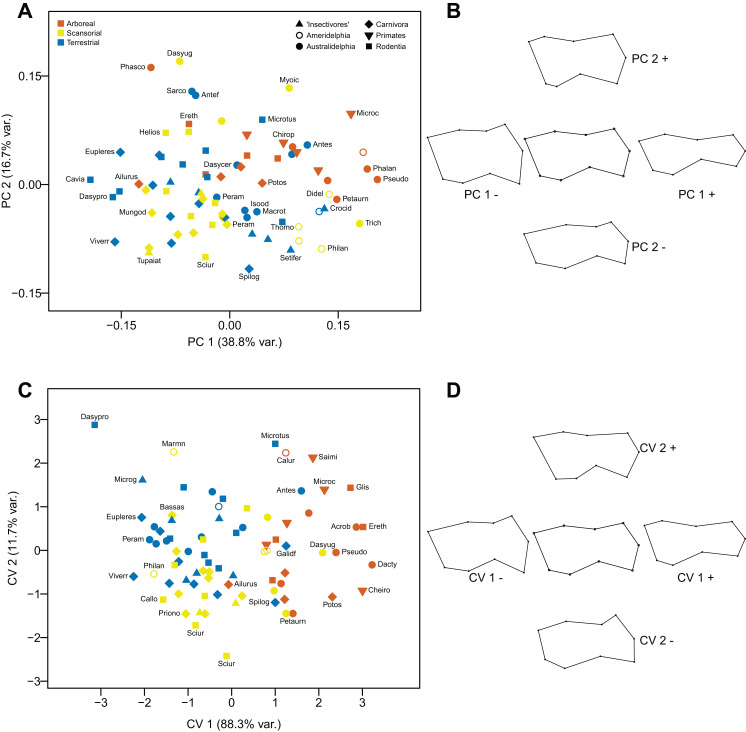
Analyses of the distal humerus (anterior view). (A) Principal components analysis. (B) Shape changes associated with each PC plus the consensus shape (center). (C) Canonical variates analysis. (D) Shape changes associated with each CV plus the consensus shape (center).

The first component (explaining 38.8% of the variance) distinguishes humeri with a distal articular surface that is long and tubular, with a rounded capitulum and an elongated and narrow trochlea (positive values), from humeri with a distal articular surface that is short and square, with an especially short and broad trochlea with a pronounced median keel (negative values). The second component (explaining 16.7% of the variance) distinguishes humeri with a short and square capitulum with a pronounced capitular tail and a fairly short and broad trochlea (positive values), from humeri with a more rectangular capitulum and a more elongated trochlea (negative values). Almost all of the arboreal taxa have positive scores on the second component, and most have positive scores on the first component: terrestrial taxa tend to have the converse pattern of scores (negative on both components) (see Figs. 7A and 7B).

#### Canonical Variates Analysis

The differences in humeral morphology along the two axes are similar to those seen in the PCA. The first axis (explaining 88.3% of the variance) separates arboreal taxa (positive scores) from terrestrial taxa (mainly negative scores): scansorial taxa lie in between these two groups on the first axis, but tend to have more negative scores on the second axis (which explains 11.7% of the variance). Marsupials group with the similar locomotor group of placentals (see Figs. 7C and 7D).

Using both Mahalanobis and Procrustes distances among groups, arboreal taxa can be distinguished from scansorial and terrestrial ones with a high degree of significance, but scansorial and terrestrial taxa cannot be distinguished from one another (even with the inclusion of the medial epicondyle). Out of the original grouped taxa, 67.5% were correctly classified (47.4% for cross-validated groups) (see Table 2).

### Proximal ulna (medial view)

#### Principal Component Analysis

**Figure 8:**
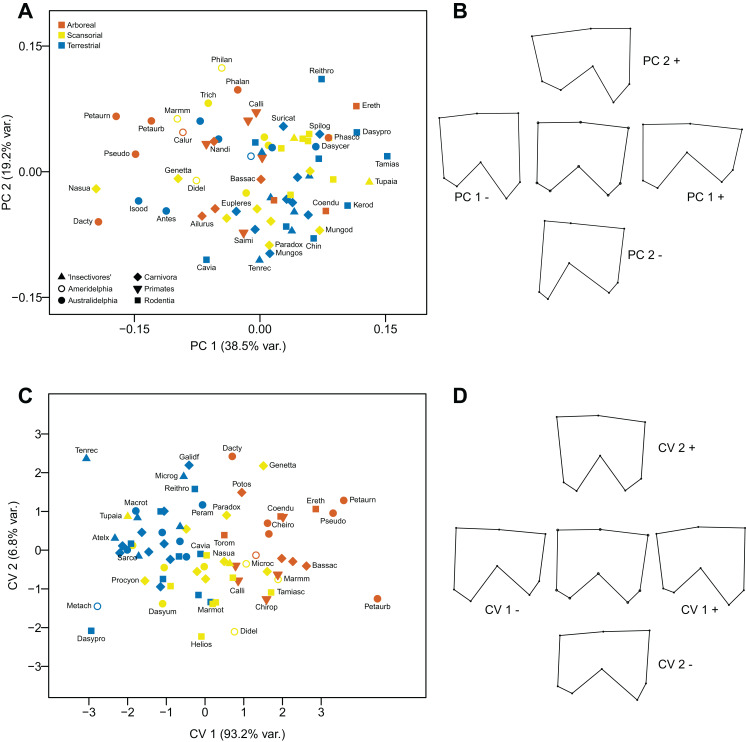
Analyses of the proximal ulna (medial view). (A) Principal components analysis. (B) Shape changes associated with each PC plus the consensus shape (center). (C) Canonical variates analysis. (D) Shape changes associated with each CV plus the consensus shape (center).

The first component (explaining 38.5% of the variance) distinguishes ulnae with a more prominent anconeal process, a less prominent and less sloping coronoid process bordering a more constrained semilunar notch, and a straighter posterior border to the ulna (reflecting a longer and straighter olecranon process) (positive values), from ulnae with an articular surface with a less prominent anconeal process and a more projecting, more sloping coronoid process bordering a more open semilunar notch, and a more curved posterior border to the ulna (reflecting a short, anteriorly-curved olecranon process) (negative values). The second component (explaining 19.2% of the variance) further reflects the morphology of the anconeal process, distinguishing ulnae with a smaller anconeal process and a more curved posterior ulna border (positive values), from ulnae with the opposite morphology (negative values). Terrestrial taxa tend to have more positive scores on the first component and arboreal taxa more negative scores, although there is much overlap (see Figs. 8A and 8B).

#### Canonical Variates Analysis

Anatomical changes along the first axis (explaining 93.2% of the variance) are essentially reversed from negative to positive values from those seen in the PCA. The anatomical differences along the second axis (explaining 6.8% of the variance) are more subtle than in the PCA, although ulnae with a more prominent anconeal process and a deeper semilunar notch have positive values. The first axis separates arboreal taxa (positive scores) from terrestrial ones (negative scores); scansorial taxa lie in between these two groups on the first axis, but tend to have more negative scores on the second axis. Marsupials group with the similar locomotor group of placentals (see Figs. 8C and 8D).

Using Mahalanobis distances among groups, the CVA distinguishes all three locomotor categories from each other high degree of significance, but using Procrustes distances only arboreal taxa can be distinguished from terrestrial ones, and neither can be distinguished from scansorial taxa. Out of the original grouped taxa, 78.4% were correctly classified (58.1% for cross-validated groups) (see Table 2).

### Proximal radius

#### Principal Component Analysis

**Figure 9:**
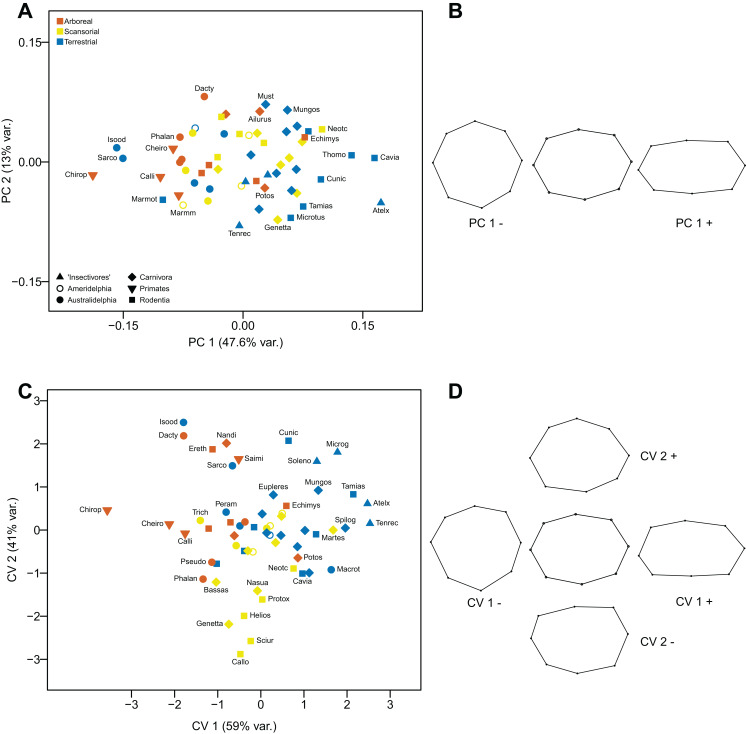
Analyses of the proximal radius (superior view). (A) Principal Components Analysis. (B) Shape changes associated with each PC plus the consensus shape (center). (C) Canonical variates analysis. (D) Shape changes associated with each CV plus the consensus shape (center).

The first component (explaining 47.6% of the variance) distinguishes radii where the proximal articular surface is ovoid in shape (positive values), from radii where the shape is round (negative values). Terrestrial taxa tend to have positive scores and arboreal ones negative scores, although there is much overlap. The second component does not explain a significant amount of the variance (see Figs. 9A and 9B).

#### Canonical Variates Analysis

The differences in proximal radial anatomy along the first axis (explaining 59% of the variance) are similar to those along the first component in the PCA. Differences along the second axis (explaining 41% of the variance) appear to relate as to whether the posterior border is rounded (positive values) or straight (negative values). The first axis largely separates terrestrial taxa (positive scores) from arboreal ones (negative scores). The scansorial taxa lie in between these two groups on the first axis, but mainly have negative scores on the second axis (see Figs. 9C and 9D).

Using Mahalanobis distances among groups, the CVA distinguishes all three locomotor categories from each other, but the distinction between scansorial taxa and the other two groups is only weakly significant: using Procrustes distances arboreal taxa are distinguished from terrestrial ones with a high degree of significance, and from scansorial ones with a weaker degree of significance: scansorial taxa cannot be distinguished from terrestrial ones. Out of the original grouped taxa, 67.8% were correctly classified (42.4% for cross-validated groups) (see Table 2).

### Proximal femur (posterior view)

#### Principal Components Analysis

**Figure 10:**
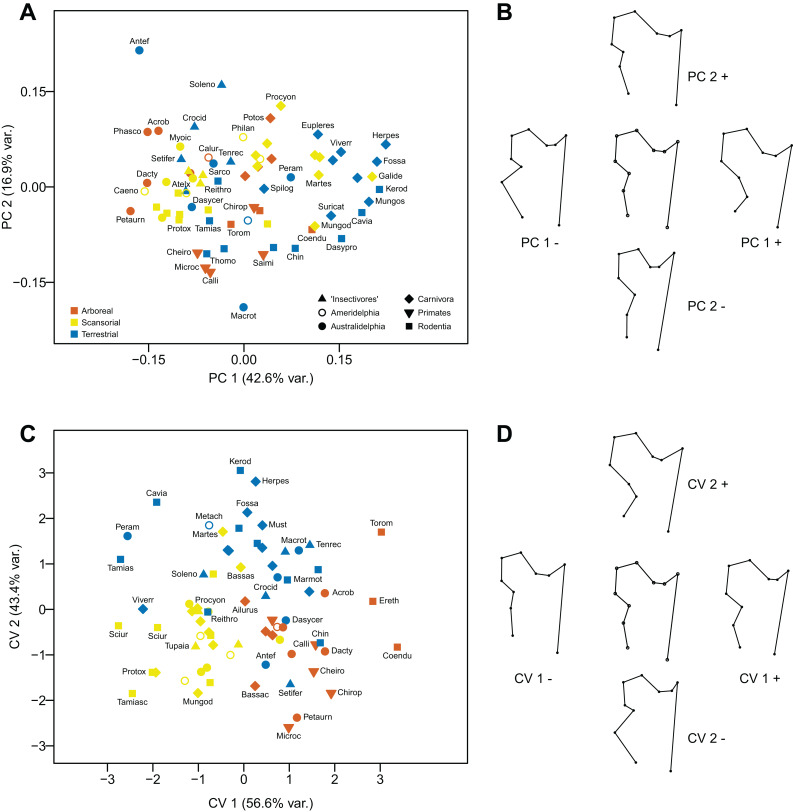
Analyses of the proximal femur (posterior view). (A) Principal components analysis. (B) Shape changes associated with each PC plus the consensus shape (center). (C) Canonical variates analysis. (D) Shape changes associated with each CV plus the consensus shape (center).

The first component (explaining 42.6% of the variance) distinguishes femora with a long femoral neck and a posteriorly (and distally) placed lesser trochanter (positive values), from femora with a short femoral neck and a medially (and proximally) placed lesser trochanter (negative values). The second component (explaining 16.9% of the variance) distinguishes femora with a short femoral neck, a somewhat medially (and proximally) placed lesser trochanter, and a greater trochanter significantly lower than the femoral head (positive values), from femora with a long femoral neck, a slightly more medially (and more distally) placed lesser trochanter, and an elevated greater trochanter that is at the same level as the femoral head. Terrestrial taxa tend to have positive scores on the first component, and arboreal taxa negative scores (see Figs. 10A and 10B).

#### Canonical Variates Analysis

This analysis picks out rather different morphological aspects of the femoral head to the PCA. The first axis (explaining 56.6% of the variance) distinguishes femora with a larger femoral head and a more medially and distally placed lesser trochanter (positive values), from femora with a small femoral head and relatively short neck with a slightly posteriorly placed lesser trochanter (negative values). The second axis (explaining 43.4% of the variance) distinguishes femora with a large femoral head, a long femoral neck, a posteriorly placed lesser trochanter, and a prominent greater trochanter (positive values), from femora with a smaller femoral head, a short femoral neck, a strongly medially-placed lesser trochanter, and a small greater trochanter. The first axis separates arboreal taxa (and many terrestrial taxa) (positive scores) from scansorial taxa (and a few terrestrial taxa) (negative scores). The second axis mainly separates terrestrial taxa (positive scores) from arboreal and scansorial ones (negatives scores). With a few exceptions, marsupials group with the similar locomotor group of placentals (see Figs. 10A and 10B).

Using Mahalanobis distances among groups, the CVA distinguishes all three locomotor categories from each other, but using Procrustes distances only arboreal taxa vs terrestrial are distinguished a high degree of significance. Out of the original grouped taxa, 81.1% were correctly classified (58.1% for cross-validated groups) (see Table 2).

### Distal femur

#### Principal Components Anaysis

**Figure 11:**
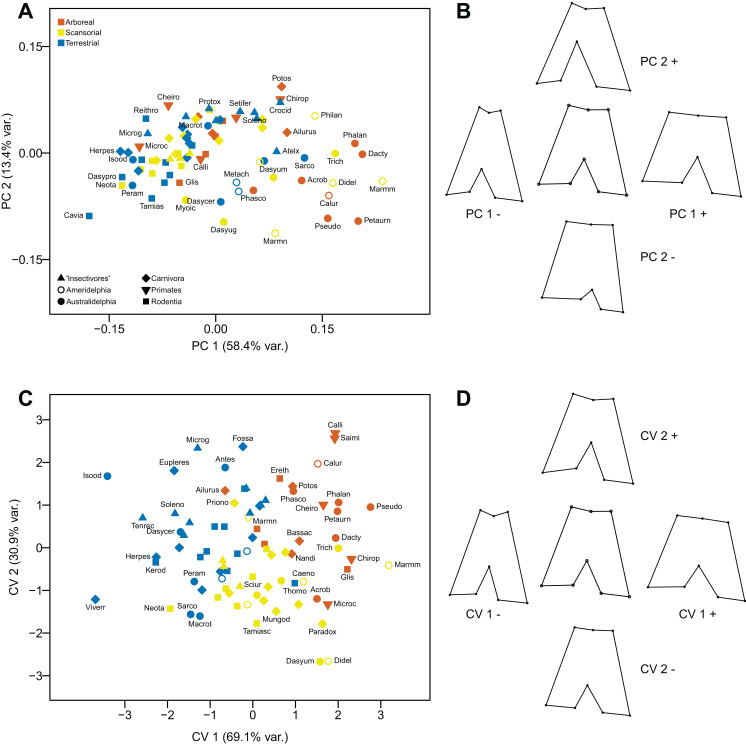
Analyses of the distal femur (inferior view). (A) Principal components analysis. (B) Shape changes associated with each PC plus the consensus shape (center). (C) Canonical variates analysis. (D) Shape changes associated with each CV plus the consensus shape (center).

The first component (explaining 58.4% of the variance) distinguishes distal femora that are short and broad, lacking an obvious patella groove, and a having broad distal end to the lateral condyle (positive values), from femora with articular surfaces that are long and narrow, with a prominent patella groove and a narrow distal end to the lateral condyle (negative values). This component primarily separates taxa on phylogenetic grounds (as previously noted, there is a marked difference between marsupials and placentals in the degree of femoral condyle asymmetry): in general, marsupials have positive scores and placentals have negatives scores. The second component (explaining 13.4% of the variance) does not distinguish the overall height of the articular surface, but femora with a moderately deep patella groove (and a relatively narrow distal portion of the articular surface), plus a relatively narrow lateral condyle and a deep incursion between the two condyles have positive values. In contrast, femora with a shallow patella groove (and a relatively broad distal portion of the articular surface), plus a relatively broad lateral condyle and a shallow incursion between the two condyles have negative values (see Figs. 11A and 11B).

#### Canonical Variates Analysis

The first axis (explaining 69.1% of the variance) distinguishes femora on similar morphological features to the first principal component, but there is less of a distinction of the symmetry of the widths of the distal condyles. The second axis (explaining 30.9% of the variance) again reflects somewhat similar morphological differences to the second principal component, but with less emphasis on distal femoral asymmetry or the depth of the incursion between the two condyles. Taxa are separated by locomotor category rather than by phylogeny: almost all arboreal taxa have positive scores on the first axis, and almost all terrestrial taxa have negative scores. The scansorial taxa lie in between these two groups, but tend to have more negative scores on the second axis. With a few exceptions, marsupials group with the similar locomotor grouping of placentals (see Figs. 11C and 11D).

Using Mahalanobis distances among groups, the CVA distinguishes all three locomotor categories from each other, but using Procrustes distances only arboreal taxa vs terrestrial are distinguished a high degree of significance. Out of the original grouped taxa, 77.1% were correctly classified (60.2 % for cross-validated groups) (see Table 2).

### Proximal tibia

#### Principal Components Analysis

**Figure 12:**
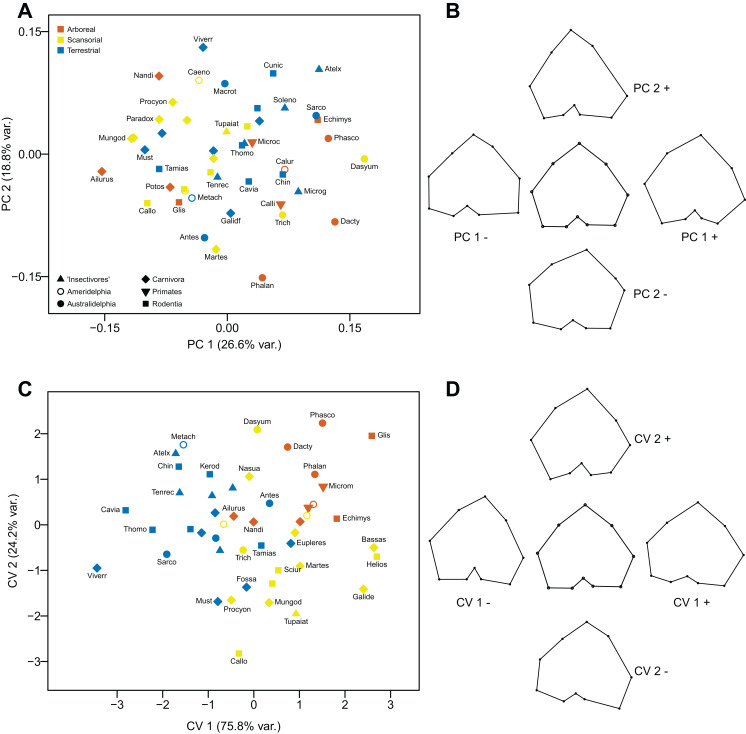
Analyses of the proximal tibia (superior view). (A) Principal components analysis. (B) Shape changes associated with each PC plus the consensus shape (center). (C) Canonical variates analysis. (D) Shape changes associated with each CV plus the consensus shape (center).

Neither component explains a high percentage of the variance, and the differences in shape are subtle, at best. The first component (explaining 26.6% of the variance) distinguishes the tibial articular surfaces primarily on the basis of the relative size and orientation of the tibial condyles, especially the lateral condyle which varies from being constricted posteriorly (positive values) to being broadened posteriorly (negative values). The second component (explaining 17.8% of the variance) distinguishes tibial articular surfaces primarily on form of the tibial tuberosity and the intercondylar notch: tibiae with a more prominent, medially-directed tuberosity and a constricted intercondylar notch (positive values), and tibiae with a less prominent, laterally-directed tuberosity and a less constricted intercondylar notch (negative values). The distribution of taxa appears to have little locomotor pattern, and instead more reflects phylogeny (see Figs. 12A and 12B).

#### Canonical Variates Analysis

The first axis (explaining 75.8% of the variance) distinguishes tibiae on the basis of slight differences in shapes of the condyles: additionally, between tibia with a shallow intercondylar notch (positive values) and tibiae with a deep intercondylar notch and a more prominent, laterally-directed tuberosity (negative values). The second axis (explaining 24.2% of the variance) distinguishes tibiae where both condyles are somewhat contracted posteriorly, and the tibial tuberosity is slightly directed laterally (positive values), from tibiae where both condyles (especially the lateral one) are expanded posteriorly (the lateral more than the medial), and the intercondylar groove is very shallow. The first axis separates the arboreal and scansorial taxa (positive scores) from most of the terrestrial taxa (with mostly negative scores). The second axis separates the arboreal taxa (positive scores) from almost all of the scansorial taxa (negative scores), and the scansorial taxa have the most negative scores on this axis. Marsupials group with the similar locomotor grouping of placentals (see Figs. 12A and 12B).

Using Mahalanobis distances among groups, arboreal forms can be distinguished from terrestrial forms with a high degree of significance, as can terrestrial forms from scansorial forms, but using Procrustes distances none of the locomotor groups can be distinguished from each other. Out of the original grouped taxa, 77.1% were correctly classified (33.3 % for cross-validated groups) (see Table 2).

## Discussion

Principal Component Analyses often showed considerable distinction among locomotor categories, although phylogeny appeared to play an important role: this was particularly true for the proximal radius and the distal femur, which largely distinguished marsupials from placentals. In contrast, the Canonical Variates Analyses were invariably able to distinguish the different locomotor groups. It is perhaps not surprising that the CVAs provided better discrimination as they are geared towards extracting the full component of features that distinguish the predefined groupings, whereas the PCAs simply show the overall variation along the axes. Thus, despite the obvious phylogenetic influence, there is a clear functional signal in long bone epiphyseal anatomy.

We did not attempt any type of phylogenetic correction of the data. This is because our future intention is to use these correlations to determine the likely locomotor behavior of “unknowns”: that is, Mesozoic mammals that are outside of the phylogenetic clusterings of crown mammals (metatherians or eutherians). Phylogenetic correction would not be appropriate for such a reference matrix; in other papers where morphometric analyses of a large sample of phylogenetically diverse extant mammals have been performed to infer the locomotor behavior of extinct ones (Chen & Wilson, 2015; Figueirido, Martín-Serra & Janis, 2016; Gould, 2014; Muñoz et al., 2017), the authors considered the phylogenetic structure of their data and results, but did not specifically apply phylogenetic correction.

With respect to the structure of the Principal Components Analyses: other papers that have analyzed joint or single bone morphology in a sample of larger-sized mammals have shown somewhat better discrimination among locomotor groups in PCAs. However, we note that the performance of such analyses appears to be related to the taxonomic level of the sample. For example, within a family, PCA usually shows good discrimination, with more or less discrete locomotor groups being discernable (e.g., Fabre et al. (2015a) for Mustelidae). However, if the taxonomic level is increased to that of the order, then there may be a “morphofunctional continuum” (sensu Chen & Wilson, 2015), but there is more overlap between groups (e.g., Panciroli et al. (2017) for Carnivora). For studies that include many orders, especially if they include both marsupials and placentals, then the groups may be almost entirely overlapping except for the highly specialized ones (Gould, 2014; Janis et al., 2020). Thus, part of the lack of discrimination in our PCAs may simply be due to the fact that we include a large taxonomic diversity in the sample.

Our poor results for the astragalus and calcaneum were both disappointing and unexpected: we did not discover a good functional locomotor signal for either bone across even different orders of placentals, let alone across all therians. It is known that the morphology of these tarsal bones is rather different between marsupials and placentals (Szalay & Sargis, 2001), but they have been shown to be good indicators of locomotor behavior within orders or families of marsupials (Bassavora, Janis & Archer, 2009; Den Boer, Campione & Kear, 2019) and placentals (Youlatos, 2003; Polly & MacLeod, 2008; Panciroli et al., 2017). While it might be possible to visually identify tarsal bones as belonging to arboreal or terrestrial mammals, based on whether the bones appear to allow for rotational movement at the ankle or restrict motion to the parasagittal plane, nevertheless quantitative analyses tend to cluster these bones according to phylogeny rather than function (Chester et al., 2015). In addition, a primary difference in the tarsal bones between locomotor types is in the relative orientation and curvatures of the their main facets (Dunn, 2018), anatomical features that would not be picked up in a two-dimensional shape analysis.

### Proximal humerus

#### Principal Component Analysis

There is an overall locomotor signal, especially within the different phylogenetic groupings, relating primarily to the degree to which the humerus is stabilized on the scapula by the rotator cuff muscles. In general, taxa with positive scores on PC1 and negative scores on PC2 are terrestrial forms with the humeroscapular motion more confined to the parasagittal plane, as indicated by the larger greater tuberosity. The larger lesser tuberosity seen in taxa with negative scores on PC1 and positive scores on PC2 may relate to the medial rotation of the humerus during use of the forelimb during active climbing (see discussion in Janis et al. (2020)); this may be the reason why it is the arboreal taxa, but not the scansorial ones, that have this combination of scores along the components.

Carnivorans in general have positive scores on PC1, but they sort out by locomotor grouping along PC2 (arboreal forms with positive scores, scansorial and terrestrial forms with mostly negative ones). Almost all of the marsupials have negative sores on PC2, but sort out by locomotor mode along PC1 (the arboreal forms having the most negative scores). Most of the terrestrial caviomorph rodents have negative scores on PC2 (*Chinchilla* (= Chin) and *Dasyprocta* (= Dasypro) being exceptions), while the arboreal and scansorial rodents mostly have positive ones. Among the “insectivores”, the scansorial scandentians have negative scores on PC2, but the terrestrial afrosoricidans and lipotyphlans have positive scores on this component (with the exception of the giant shrew *Crocidura* (= Crocid)). Primates have negative scores on PC1 and positive scores on PC2 (with the exception of the saki, *Chiropotes* (= Chirop), with a positive score on PC1) (see Figs. 6A and 6B).

#### Canonical Variates Analysis

The first axis has a strong locomotor signal, but arboreal and terrestrial taxa are both widely scattered on the second axis, with no apparent correlation with phylogeny. The preponderance of arboreal taxa with negative scores on the first axis resembles their distribution in the PCA, reflecting the possession of a larger lesser tuberosity and a smaller greater tuberosity. The majority of the scansorial taxa have negative scores on the second axis, but this does not distinguish them from the other two locomotor groups. The arboreal carnivorans have the among the least negative scores out of the arboreal forms, clustering with a few of the terrestrial carnivorans along this axis. Arboreal forms with positives scores on the first axis include the possum *Phalanger* (= Phalan) and the caviomorph rodent *Coendu*. A couple of scansorial taxa have highly positive scores on the first axis, clustering among the terrestrial taxa: the carnivoran *Genetta* and the quoll *Dasyurus maculatus* (= Dasyum) (see Figs. 6C and 6D).

### Distal humerus

#### Principal Components Analysis

There is distinct locomotor signal along PC1, similar to that found along the first component in the PCA of Figueirido, Martín-Serra & Janis (2016), distinguishing between humeral anatomy allowing for a high degree of rotation at the elbow joint in arboreal taxa (positive scores), from that restricting the amount of rotation and providing a greater degree of forelimb stability with the forelimb locked into a prone position in terrestrial ones (negative scores). An extremely elongated and narrow trochlea is especially prominent in arboreal marsupials. Our results here reflect this combination of functional and phylogenetic distinction: taxa with high positive scores on PC1 are almost exclusively arboreal and scansorial marsupials (plus a couple of terrestrial marsupials), such as the dasyurid *Antechinus* (=Antes) and the didelphid *Metachirus* (= Metach), and also lemuriform primates). Other primates and, with a few exceptions, arboreal carnivores and rodents have lower positive scores, as do the peramelid marsupials (all terrestrial). Taxa with negative scores on PC1 are primarily terrestrial and scansorial carnivorans and rodents. Some anomalies exist: some terrestrial carnivorans (*Spilogale* (= Spilo)) and rodents (*Microtus* and *Thomomys* (= Thomo) have positive scores on PC1, clustering with the arboreal taxa. Conversely, some arboreal forms have negative scores, clustering with the terrestrial taxa (e.g., the carnivoran *Ailurus*, the caviomorph rodent *Erithizion* (= Erith), and the dasyurid marsupial *Phascogale* (= Phasco)). The terrestrial “insectivores” are scattered along PC1 (but all with negative scores on PC2) (see Figs. 7A and 7B).

#### Canonical Variates Analysis

The taxa that fall in the exclusively arboreal portion of the first axis (positive scores) are, as with the proximal humerus, primarily primates and arboreal marsupials, and some of the arboreal rodents. With the exception of *Potos*, the arboreal carnivorans have less positive scores, falling close to the overlap with the terrestrial taxa. Terrestrial taxa falling within the confines of the arboreal area along PC1 are mostly carnivorans (the highest positive scores belonging to *Spilogale* and *Galidictis* (= Galidf)), plus the rodent *Microtus* and the dasyurid marsupial *Antechinus* (= Antes). Both *Microtus* and the terrestrial caviomorph rodent *Dasyprocta* are notable outliers on the positive side of the second axis (see Figs. 7C and 7D).

### Proximal ulna (medial view)

#### Principal Components Analysis

There is a considerable scatter of locomotor modes along PC1, but in general arboreal taxa have negative scores, with an ulna morphology reflecting considerable rotational movement of the forearm around the humeroulnar joint. The exceptions here are the arboreal caviomorph rodents and the dasyurid marsupial *Phascogale*, with positive scores. With a few exceptions (e.g., the scansorial procyonid carnivoran *Nasua* (although note that this taxon is not an outlier on the CVA)), the taxa with the highest negative scores are arboreal and scansorial marsupials, and terrestrial marsupials also tend to have negative scores (e.g., the bandicoot *Isoodon* (= Isood)), demonstrating an effect of phylogeny as well as locomotion. In contrast, most scansorial and terrestrial placentals have positive scores on PC1 (a notable exception being the caviomorph rodent *Cavia*) (see Figs. 8A and 8B).

#### Canonical Variates Analysis

Although the overlap between scansorial forms and the other locomotor groups is considerable, there is little overlap between terrestrial and arboreal forms. The arboreal forms with the highest positive scores along the first axis are mainly the marsupials (especially *Petaurus breviceps* (= Petaurb)), although the striped possum *Dactylopsila* (= Dacty) has low positive scores, as do a couple of primates (e.g., *Microcebus* (= Microc) and *Callithrix* (= Calli)). Terrestrial taxa with very high negative scores include the afrosoricidan *Tenrec* (with a high positive score on the second axis), and the didelphid marsupial *Metachirus* and the caviomorph rodent *Dasyprocta* (both with high negative scores on the second axis) (Figs. 8C and 8D).

### Proximal radius

#### Principal Components Analysis

A round radial head, permitting rotation of the forelimb around the elbow joint is well known to distinguish arboreal taxa from terrestrial ones (which have an ovoid radial head, restricting forelimb rotation) (MacLeod & Rose, 1993). Here, perhaps because we have added marsupials to the mix of taxa, this PCA reveals a strong phylogenetic component, and there is little functional signal. All of the marsupials have negative scores on the first axis, perhaps reflecting the need for rotation of the forelimb in early ontogeny (or alternatively simply reflecting the retention of a more conservative type of forelimb anatomy). Within the array of marsupials in the morphospace there appears to be no correlation with locomotor mode: the highest negative scores along PC1 belong to terrestrial australidelphids, the bandicoot *Isoodon* and the dasyurid *Sarcophilus* (= Sarco). The placentals with high negative scores on this component are primarily primates (and, strangely, the terrestrial sciuromorph rodent *Marmota* (= Marmot)). Taxa with positive scores on PC1 are primarily terrestrial and scansorial rodents and carnivorans, and the arboreal members of these groups tend to have more negative scores (with the notable exception of the caviomorph rodent *Echimys*) (see Figs. 9A and 9B).

#### Canonical Variates Analysis

Although there is a large overlap between the arboreal and terrestrial taxa, a locomotor signal is apparent. Those arboreal taxa that have highly negative scores on the first axis are primates and the arboreal phalangeriform marsupials, and the terrestrial taxa with highly positive scores on this axis are primarily lipotyphlan and afrosoricid “insectivores.” The peramelid marsupials, which usually cluster near to one other in our analyses, show widely divergent anatomies of the radial head. *Macrotis* (the bilby, = Macrot) has one of the most positive scores on the first axis, clustering with the terrestrial placentals: but one of the bandicoots (*Isoodon*) has high negative scores, while the other (*Perameles* (= Peram)) falls within the overlap of the three locomotor groups. The second axis potentially distinguishes scansorial taxa from the other locomotor groups. Taxa with highly negative scores are primarily squirrels (sciuromorph rodents), but also the carnivoran *Genetta*, and the scansorial carnivorans *Nasua* and *Bassariscus* (= Bassas) (see Figs. 9C and 9D).

### Proximal femur (posterior view)

#### Principal Components Analysis

The first component distinguishes between taxa where the hindlimb motion is more restricted to the parasagittal plane (positive scores) from those where there is a greater degree of rotation of the hip joint (negative scores). Although PC1 does largely reflect locomotor type, almost all of the marsupials have negative scores on this component, the bandicoot *Perameles* being the exception. However, within the marsupials, the arboreal and scansorial taxa have the most negative scores on PC1 (with the exception of the diminutive terrestrial dasyurid *Antechinus* (= Antef)), sharing this area of the first component with (largely) scansorial sciuromorph rodents and scandentians, but also with the terrestrial “insectivores” (the latter also having positive scores on PC2). Interestingly, the primates do not have highly negative scores on PC1, with some of the anthropoids (e.g., *Saimiri* (= Saimi)) even having weakly positive scores. In the positive portion PC1 the carnivorans and caviomorph rodents largely sort out along an arboreal plus scansorial (lower scores) to a terrestrial (higher scores) axis. PC2 does not appear to distinguish taxa on either locomotor mode or phylogeny (although the leaping primates do have low scores) (see Figs. 10A and 10B).

#### Canonical Variates Analysis

There is quite a large overlap between terrestrial and arboreal taxa along the first axis, although the arboreal rodents have the highest positive sores. The highest negatives scores belong to either terrestrial forms (rodents (*Cavia* and *Tamias*), the carnivoran *Viverricula* (= Viverr), and the bandicoot *Perameles*), which also have positive scores on the second axis, and scansorial sciuromorph rodents, which also have negatives scores on the second axis. Arboreal primates and carnivorans (plus some arboreal marsupials) tend to fall in the overlap between the terrestrial and arboreal taxa on the first axis. The second axis shows a somewhat clearer separation between terrestrial taxa (mostly positive scores) and scansorial plus arboreal taxa (mostly negative scores). The main terrestrial outliers on the second axis (with high negative scores) are the rodent *Chinchilla*, the dasyurid *Antechinus* and the “insectivore” *Setifer* and the arboreal outlier (with a high positive score) is the rodent *Toromys* (= Torom) (see Figs. 10C and 10D).

### Distal femur

#### Principal Component Analysis

Taxa are separated primarily by phylogeny. With the exception of the peramelids (the only cursorial quadrupedal marsupials included here), and the dasyurid *Myoictis* (= Myoic), all of the marsupials have positive scores on PC1, and all of the taxa with scores of ~0.14 or greater are marsupials (*Sarcophilus* being the only terrestrial taxon among them). *Phascogale*, the sole arboreal dasyurid, is the only arboreal marsupial not in this grouping. In the more negative portion of PC1, occupied mainly by placentals, the arboreal taxa have positive (or only slightly negative) scores (reflecting a femoral morphology less restricted to parasagittal motion): the terrestrial taxa in general have the most negative scores, and the scansorial taxa fall in the middle. The exceptions are as follows: the terrestrial “insectivorans” *Solenodon* (= Soleno), *Setifer*, *Atelerix* (= Atelx) and *Crocidura* have positive scores and cluster with the arboreal placentals, and the lemuriform primates (*Microcebus* and *Cheirogaleus* (= Cheiro)) have highly negative scores, clustering with the terrestrial placentals. The case of the lemuriforms is more easily explained: these little primates move from tree to tree by leaping, and their femoral morphology reflects a relatively restricted parasagittal motion of the lower limb on the femur.

Along PC2, the arboreal marsupials have almost entirely negative scores, but among the placentals the negative scores belong mainly to the terrestrial and scansorial taxa. However, this component does separate, to a large extent, carnivorans (with positive scores) from terrestrial and scansorial rodents (especially caviomorphs) (with negative scores) (see Figs. 11A and 11B).

#### Canonical Variates Analysis

There is a moderate degree of overlap between the arboreal taxa (positive scores) and the terrestrial ones (negative scores) along the first axis: the main taxa with anomalous placements being the arboreal carnivoran *Ailurus* (with a negative score) and terrestrial rodent *Thomomys* (with a positive score). The highest scores on the first axis belong to arboreal and scansorial marsupials, and to primates, with the arboreal caviomorph rodents having the lowest positive scores. The bandicoot *Isoodon* and the carnivoran *Viverricula* are outliers on the first axis with highly negative scores. On the second axis, all of the scansorial taxa have negative scores, with the exception of the didelphid marsupial *Marmosops* (= Marmn) and the carnivoran *Prionodon* (= Priono). However, both arboreal and terrestrial taxa span positive to negative scores on the second axis, and there does not appear to be any influence of phylogeny to the pattern of their distribution.

### Proximal tibia

#### Principal Components Analysis

The separation of taxa here largely reflects phylogeny. Almost all of the carnivorans all have negative scores on PC1, while the marsupials have mostly positive scores. The caviomorph rodents all have positive scores, while the other rodents mostly have negative ones. The “insectivorans” and the primates all have scores that are around zero or weakly positive. There appears to be little in the way of functional or phylogenetic pattern to the placement of taxa on the second component.

#### Canonical Variates Analysis

Despite the relatively poor resolution of this analysis in terms of distinguishing locomotor mode, it is nonetheless interesting as this is the only analysis where the scansorial taxa are not positioned in between the arboreal and terrestrial ones on the first axis. Here, the scansorial taxa occupy the same part of the positive side of the first axis as the arboreal ones, and indeed are the ones with the most positive scores, and the arboreal taxa are restricted to the positive side of the second axis. Two arboreal carnivorans (*Ailurus* and *Nandinia* (= Nand)) have negative scores on the first axis, falling into the area of overlap between terrestrial and scansorial taxa, while the terrestrial carnivoran *Eupleres* has a positive score. *Ailurus* (the red panda) also clustered with terrestrial taxa in the CVAs for the femora and the distal humerus. Unlike the condition in many of the other analyses the terrestrial “insectivorans” all cluster with the other terrestrial taxa and, the carnivoran *Viverricula* is an outlier with highly negative scores. The scansorial taxa mostly have negative scores on the second axis, and include the taxa with the most negative scores.

## Conclusions

Our results show that long bone epiphyseal anatomy (i.e., the joint articular surfaces) of small therian mammals, the type of postcranial remains that are the most likely to be preserved as fragmentary fossils, can indeed be used as indicators of locomotor mode. While the Principal Components Analyses often reflected phylogeny, a functional signal was apparent in most cases, and the Canonical Variates Analyses usually showed discrimination as to locomotor mode. All of the long bone elements were able to distinguish arboreal from terrestrial taxa, but they varied in their abilities to distinguish scansorial taxa from either arboreal or terrestrial ones.

Table 4 summarizes the results of the Canonical Variates Analyses, and Table 5 provides a summary of how the epiphyseal anatomy differs between the different locomotor types. Interestingly, the more proximal elements performed better than the more distal ones. In particular, the proximal femur had the best ability to correctly reclassify taxa (80%) and was able to distinguish among the three locomotor modes in both Mahalanobis and Procrustes distances analyses, being the only element that could always distinguish between scansorial and terrestrial taxa. Although the proximal humerus was inferior at correctly reclassifying taxa (71%), and also inferior in comparison with some of the more distal forelimb elements, it was more reliable in its ability to distinguish scansorial taxa than those elements.

**Table 5:** Summary of the differences in morphology of the limb epiphyses considered here in taxa of different locomotor mode.

Element	Arboreal taxa	Scansorial	Terrestrial taxa
Proximal humerus	Small greater tuberosity	Intermediate morphology, but have small lesser tuberosity	Small greater tuberosity
	May have large lesser tuberosity		Small lesser tuberosity
	Round humeral head		Ovoid humeral head
Distal humerus	Round capitulum, without capitular tail	Intermediate morphology, but more like terrestrial taxa	Large, square capitulum with capitular tail
	Long, tubular trochlea		Shorter trochlea with strong medial wall and distal projection
Proximal ulna	Anconceal process not prominent	Intermediate morphology, but more like terrestrial taxa	Anconceal process prominent
	Sloping and projecting coronoid process		Less prominent and sloping coronoid process
	More open semilunar notch		More constrained semilunar notch
	Curved posterior border		Straight posterior border
Proximal radius	Circular in shape	Intermediate morphology, but more like arboreal taxa	Oval or even rectangular in medio-lateral direction
Proximal femur	Short femoral neck	Intermediate morphology, but more like terrestrial taxa	Long femoral neck
	Small greater trochanter		Large greater trochanter
	Large lesser trochanter placed medially and proximally		Smaller lesser trochanter placed posteriorly and distally
Distal femur	Short and broad	Intermediate morphology, but more like terrestrial taxa	Long and narrow
	Shallow intercondylar groove		Deep intercondylar groove
	Shallow patella groove		Deep patella groove
Proximal tibia	Less prominent tibial tuberosity	Intermediate morphology, but more like arboreal taxa	More prominent tibial tuberosity
	More shallow intercondylar groove		Deeper intercondylar groove

In contrast, the more distal limb elements, especially the tarsal bones but also the proximal radius and the proximal tibia, had relatively poorer performances than the more proximal ones. We note that we were not able to obtain as many images for these elements, which may have resulted in less statistical significance, but it was also apparent to us from looking at the bones themselves that these elements were not as informative as the more proximal ones, at least in these small mammals. In contrast, for example, the shape of the proximal tibia may be highly informative about locomotor mode in larger mammals (Ercoli, Prevosti & Álvarez, 2012), but we did not see a similar extent of morphological variation in the small mammals; perhaps this is because none of them are cursorial, with a knee joint more restricted to motion in the parasagittal plane.

It may be the case that the more proximal elements are the more reliable indicators across a broad taxonomic diversity because they reflect both the weight-bearing capacity of the limb, and the manouverablity of the limb across a range of postures. In contrast, the joint morphology of the more distal elements may reflect more precise aspects of limb positioning, and so may be more subjected to phylogenetic differences. This would be especially the case for the tarsal bones, as intertarsal mobility is known to have evolved convergently in different groups of mammals, in both therians and nontherians (Jenkins & Krause, 1983).

The hindlimb elements appear to be superior at distinguishing scansorial forms (especially between scansorial and terrestrial taxa) to the forelimb ones. These differences between forelimbs and hindlimbs may reflect different adaptive pressures: the role of the hindlimb is mainly propulsive, while that of the forelimb is more reflective of support, absorption of impact, and other behaviors such as reaching in arboreal forms.

It is not our intention here to make recommendations for which bone in particular to use to determine the locomotor behavior of an extinct small mammal. We note that if a choice of elements were available for any particular individual, then there would also likely be more complete skeletal elements (e.g., complete long bones) that would be more informative in their relative proportions (Chen & Wilson, 2015). The strength of results of these analyses is that they provide the opportunity to make a determination for a single preserved element, or a cluster of different elements, from fossils that might not be classifiable even to the level of metatherian or eutherian. However, we can make some cautionary comments, noting in particular that tarsal elements unfortunately do not provide a good signal at this level, despite the fact that they may be excellent elements for determining locomotor behavior if the taxon can be identified to order (see Chester et al. (2015), with respect to primates). We must also emphasize that these results are only applicable to therian mammals: the posture of non-therians, and their joint articulatory surfaces (especially the elbow joint, see Jenkins (1973)) are sufficiently different to preclude confident assessment of locomotor mode from a therian reference.

**Figure 13:**
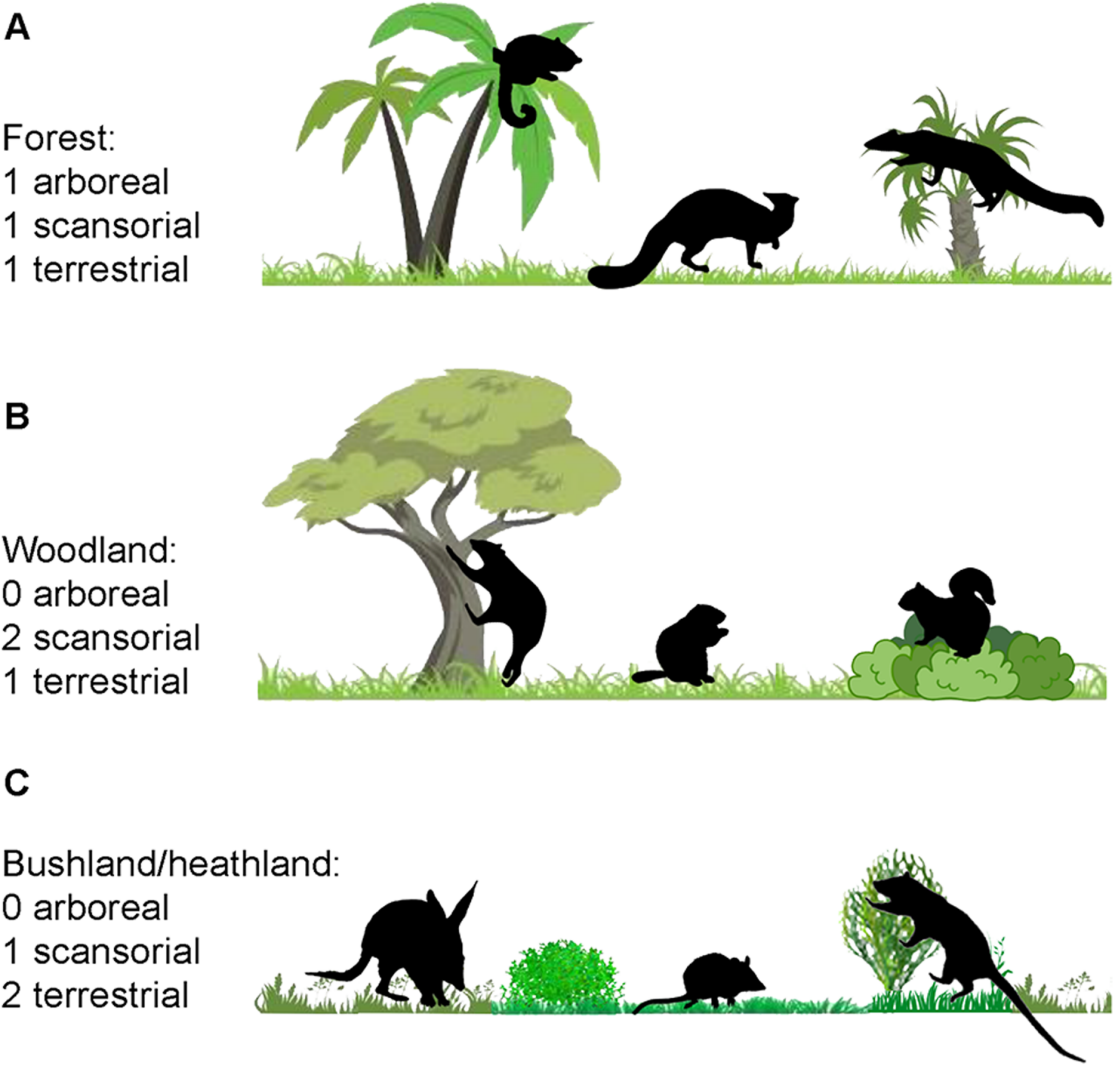
Scenarios to illustrate how fragmentary limb bone elements might be used to determine the locomotor composition of a local fauna, and hence the nature of the habitat. (A) Tropical forest (Madagascan scene): greater mouse lemur *(Cheirogaleus major*), arboreal; Eastern falanouc (*Eupleres goudotti*), terrestrial; ring-tailed vontsira (*Galidia elegans*), scansorial. (B) Temperate woodland (North American scene): raccoon (*Procyon lotor*), scansorial; marmot (*Marmota monax*), terrestrial; Eastern gray squirrel (*Sciurus carolinensis*), scansorial. (C) Temperate bushland/heathland (Australian scene): greater bilby (*Macrotis lagotis*), terrestrial; yellow-footed antechinus (*Antechinus flavipes*), terrestrial; spotted-tail quoll (*Dasyurus maculatus*), scansorial. The images of the vegetation are obviously generic. The taxa figured are shown only approximately to scale, and do not necessarily coinhabit in the same exact local: these scenarios are for illustrative (and entertainment) purposes only. All images of vegetation from Webstockreview.net, all images of mammals from phylopic.org (images available for reuse under the Public Domain Dedication 1.0 license, or Creative Commons Attribution 3.0 unported license https://creativecommons.org/licenses/by/3.0/). Image credits: *C. major* by Maky, Gabriella Skollar, and Rebecca Lewis; *Eupleres goudotii* and *G.elegans* by Margot Michaud; *Procyon lotor* by Mathieu Basille; *M. monax* by T. Michael Keesey; *S. carolinensis* by Anthony Caravaggi; *M. lagotis* and *D. maculatus* by Sarah Werning; *A. flavipes* by Robbi Bishop-Taylor.

Our results hold promise for the determination of past habitat types from assemblages of small mammal fragmentary postcrania: while taxonomic assignation of these remains may be difficult (although they can usually be identified as therian versus non-therian), the taxon-free diversity of locomotor modes in a fossil assemblage may provide information on ancient habitats. Future work will address such issues, but meanwhile Figure 13 provides a glimpse of how bone fragments from small mammal assemblages might provide reliable information on the environments that they inhabited.

## Supplemental Information

10.7717/peerj.9634/supp-1 Supplemental Information 1 Phylogenetic tree for the species used in this study. Obtained from Vertlife.org (Upham, Esselstyn & Jetz, 2019).

10.7717/peerj.9634/supp-2 Supplemental Information 2 Details of marsupial species used in analyses. Key to museum abbreviations: AMNH = American Museum of Natural History (New York); FMNH = Field Museum of Natural History (Chicago); MCZ = Museum of Comparative Zoology (Harvard University): UCMP = University of California Museum of Paleontology (Berkeley).

10.7717/peerj.9634/supp-3 Supplemental Information 3 Details of placental species used in analyses: primates and insectivorous mammals. Key to museum abbreviations as in Table S1.

10.7717/peerj.9634/supp-4 Supplemental Information 4 Details of placental species used in analyses: rodents. Key to museum abbreviations as in Table S1.

10.7717/peerj.9634/supp-5 Supplemental Information 5 Details of placental species used in analyses: carnivorans. Key to museum abbreviations as in Table S1.

10.7717/peerj.9634/supp-6 Supplemental Information 6 Description of 2D landmarks digitized on each bone epiphysis analyzed.

10.7717/peerj.9634/supp-7 Supplemental Information 7 Results of the phylogenetic Procrustes ANOVAs for each bone epiphysis. Log CS = log-transformed centroid size. Locomo. = locomotor categories. Df = degrees of freedom. SS = sum of squares. MS = mean squares. Rsq = R squared. F = F statistic. Z = Z score (effect size). Pr(>F) = p-value obtained from permutation. Values with significance levels <0.05 are in bold face type.

10.7717/peerj.9634/supp-8 Supplemental Information 8 Procrustes coordinates for each taxon by each bone.

## Additional Information and Declarations

### Data Availability

The following information was supplied regarding data availability: The Procrustes coordinates for each bone for each taxon, which were derived from the geometric morphometric analyses and form the bases of the statistical analyses, are available in Table S7.
