# Supplementary figures and images for: Postcranial elements of small mammals as indicators of locomotion and habitat
Source: PeerJ. 2020 Sep 2;8:e9634. doi: 10.7717/peerj.9634 (PMC7474524; doi:10.7717/peerj.9634)

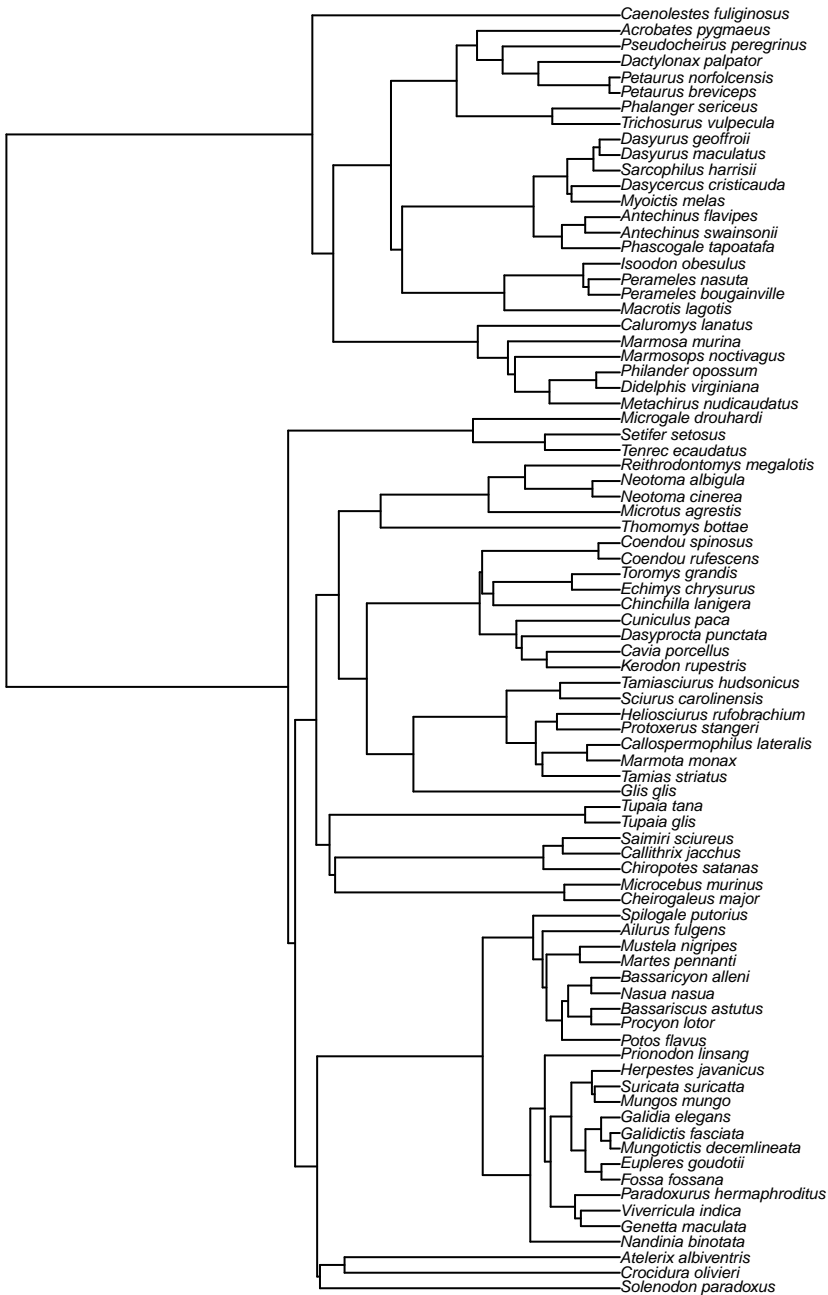

Supplement: Supplemental Information 1 — Obtained from Vertlife.org (Upham, Esselstyn & Jetz, 2019). [file peerj-08-9634-s001.pdf]
