# Supplementary material for: Postcranial elements of small mammals as indicators of locomotion and habitat
Source: PeerJ. 2020 Sep 2;8:e9634. doi: 10.7717/peerj.9634 (PMC7474524; doi:10.7717/peerj.9634)
Supplement: Supplemental Information 2 — Key to museum abbreviations: AMNH = American Museum of Natural History (New York); FMNH = Field Museum of Natural History (Chicago); MCZ = Museum of Comparative Zoology (Harvard University): UCMP = University of California Museum of Paleontology (Berkeley). [file peerj-08-9634-s002.docx]

| Order | Family | Taxon | Spec.# | Common name | Abbr. | Loc. | PH | DH | PU | PR | PF | DF | PT |
| --- | --- | --- | --- | --- | --- | --- | --- | --- | --- | --- | --- | --- | --- |
| **Ameridelphia** |  |  |  |  |  |  |  |  |  |  |  |  |  |
| Didelphimorphia | Caenolestidae | *Caenolestes fuliginosus* | AMNH 64498 | silky shrew opossum | Caeno | S | X | X |  | X | X | X | X |
|  | Didelphidae | *Caluromys lanatus* | MCZ 37857 | brown-eared woolly opossum | Calur | A | X | X | X |  | X | X | X |
|  |  | *Didelphis virginiana* | MCZ 62199 | Virginia opossum | Didel | S | X | X | X | X | X | X | X |
|  |  | *Marmosa murina* | AMNH 133211 | murine mouse opossum | Marmm | S |  |  | X | X |  | X |  |
|  |  | *Marmosops noctivaga* | MCZ 37860 | white-bellied slender mouse opossum | Marmn | S | X | X |  |  | X | X |  |
|  |  | *Metachirus nudicaudatus* | AMNH 266499 | brown four-eyed opossum | Metach | T | X | X | X |  |  | X | X |
|  |  | *Metachirus nudicaudatus* | MCZ 167 | brown four-eyed opossum | Metach | T |  |  |  | X | X | X |  |
|  |  | *Philander opossum* | AMNH 254509 | grey four-eyed opossum | Philan | S | X | X | X |  | X | X |  |
| **Australidelphia** |  |  |  |  |  |  |  |  |  |  |  |  |  |
| Dasyuromorphia | Dasyuridae | *Antechinus swainsonii* | AMNH 65718 | dusky antechinus | Antes | T |  | X | X | X |  | X | X |
|  |  | *Antechinus flavipes* | MCZ 65718 | yellow-footed antechinus | Antef | T |  | X |  |  | X |  |  |
|  |  | *Antechinus* sp. | MCZ 58145 | antechinus | Ante |  | X | X |  |  |  |  |  |
|  |  | *Dasycercus cristicauda* | MCZ 558147 | crested-tail mulgara | Dasycer | T | X | X | X |  | X | X |  |
|  |  | *Dasyurus geoffroii* | MCZ 6932 | Western quoll | Dasyug | S | X | X | X |  | X | X |  |
|  |  | *Dasyurus maculatus* | AMNH 66162 | spotted-tail quoll | Dasyum | S | X | X | X | X | X | X | X |
|  |  | *Myoictis melas* | AMNH 221649 | three-striped dasyure | Myoic | S | X | X | X | X | X | X |  |
|  |  | *Phascogale tapoatafa* | AMNH 160267 | brush-tailed phascogale | Phasco | A | X | X | X |  | X | X |  |
|  |  | *Sarcophilus harrisii* | AMNH 65672 | Tasmanian devil | Sarco | T |  | X | X | X | X | X | X |
|  |  | *Sarcophilus harrisii* | AMNH 35634 | Tasmanian devil | Sarco | T | X |  |  |  |  |  |  |
| Diprotodontia | Acrobatidae | *Acrobates pygmaeus* | MCZ 29144 | pygmy feather-tailed glider | Acrob | A | X | X |  |  | X | X |  |
|  | Petauridae | *Dactylonax palpator* | AMNH 194579 | long-fingered triok (striped possum) | Dacty | A | X | X | X | X | X | X | X |
|  |  | *Petaurus breviceps* | MCZ 58144 | sugar glider | Petaurb | A |  |  | X |  |  |  |  |
|  |  | *Petaurus norfolcensis* | AMNH 35763 | squirrel glider | Petaurn | A | X | X | X | X | X | X |  |
|  | Phalangeridae | *Phalanger sericeus* | AMNH 191203 | silky cuscus | Phalan | A | X | X | X | X | X | X | X |
|  |  | *Trichosurus vulpecula* | AMNH 35708 | common brush-tailed possum | Trich | S | X | X | X | X | X | X | X |
|  | Pseudocheiridae | *Pseudocheirus peregrinus* | UCMP 82342 | common ringtail possum | Pseudo | A | X | X | X | X |  | X |  |
|  |  | *Pseudocheirus peregrinus* | FMNH 134502 | common ringtail possum | Pseudo | A |  |  | X |  |  |  |  |
| Peramelemorphia | Peramelidae | *Isoodon obesulus* | UCMP 77305 | Southern brown bandicoot | Isood | T | X | X | X | X |  | X |  |
|  |  | *Perameles nasuta* | AMNH 65659 | long-nosed bandicoot | Peram | T | X | X | X | X | X | X |  |
|  |  | *Perameles bougainville* | MCZ 52970 | Western barred bandicoot | Perab | T |  | X |  |  |  |  |  |
|  | Thylacomylidae | *Macrotis lagotis* | AMNH 35685 | greater bilby | Macrot | T | X | X | X | X | X | X | X |
