# Supplementary material for: Postcranial elements of small mammals as indicators of locomotion and habitat
Source: PeerJ. 2020 Sep 2;8:e9634. doi: 10.7717/peerj.9634 (PMC7474524; doi:10.7717/peerj.9634)
Supplement: Supplemental Information 3 — Key to museum abbreviations as in Table S1. [file peerj-08-9634-s003.docx]

| Order | Family | Taxon | Spec.# | Common name | Abbr. | Loc. | PH | DH | PU | PR | PF | DF | PT |
| --- | --- | --- | --- | --- | --- | --- | --- | --- | --- | --- | --- | --- | --- |
|  |  |  |  |  |  |  |  |  |  |  |  |  |  |
| Afrosoricida | Tenrecidae | *Microgale drouhardi* | MCZ 46017 | Drouhard’s shrew tenrec | Microg | T | X | X | X | X |  | X | X |
|  |  | *Setifer setosus* | MCZ 5112 | greater hedgehog tenrec | Setifer | T | X | X | X |  | X | X | X |
|  |  | *Setifer setosus* | AMNH 170532 | greater hedgehog tenrec | Setifer | T |  |  |  |  |  | X |  |
|  |  | *Tenrec ecaudatus* | MCZ 44967 | common or tailess tenrec | Tenrec | T | X | X |  |  | X | X | X |
|  |  | *Tenrec ecaudatus* | AMNH 212914 | common or tailess tenrec | Tenrec | T |  |  | X | X |  | X |  |
|  |  |  |  |  |  |  |  |  |  |  |  |  |  |
| Eulipotyphla | Erinaceidae | *Atelerix albiventris* | MCZ 60743 | African pygmy hedgehog | Atelx | T | X | X | X | X | X | X | X |
|  | Solenodontidae | *Solenodon paradoxus* | MCZ 12380 | Hispaniolan solenodon | Soleno | T | X | X |  | X |  | X | X |
|  |  | *Solenodon paradoxus* | AMNH 119581 | Hispaniolan solenodon | Soleno | T |  |  | X |  |  | X |  |
|  | Soricidae | *Crocidura olivieri* | AMNH 239321 | African giant shrew | Crocid | T | X | X | X |  | X | X |  |
|  |  |  |  |  |  |  |  |  |  |  |  |  |  |
| Scandentia | Tupaiidae | *Tupaia glis* | AMNH 55561 | common tree shrew | Tupaiag | S | X | X |  |  | X | X |  |
|  |  | *Tupaia tana* | AMNH 35921 | large tree shrew | Tupaiat | S | X | X | X |  | X |  | X |
|  |  | *Tupaia* sp. | UCMP 123674 | tree shrew | Tupaia | S |  |  |  |  |  | X |  |
|  |  |  | AMNH 215174 | tree shrew | Tupaia | S | X |  | X |  | X | X |  |
|  |  |  |  |  |  |  |  |  |  |  |  |  |  |
| Primates | Cheirogaleidae | *Cheirogaleus major* | AMNH 110640 | greater dwarf lemur | Cheiro | A | X | X | X | X | X | X |  |
| (Lemuriformes) |  | *Microcebus murinus* | AMNH 185627 | gray dwarf lemur | Microc | A | X | X | X |  | X | X | X |
|  |  |  |  |  |  |  |  |  |  |  |  |  |  |
| Primates | Callitrichidae | *Callithrix jacchus* | MCZ 439 | common marmoset | Calli | A | X | X | X | X | X | X | X |
| (Anthropoidea) | Cebidae | *Saimiri sciureus* | MCZ 4247 | squirrel monkey | Saimi | A | X | X | X | X | X |  |  |
|  | Pithecidae | *Chiropotes satanas* | MCZ 6082 | black-bearded saki | Chirop | A | X | X | X | X | X | X |  |
