# Supplementary material for: Postcranial elements of small mammals as indicators of locomotion and habitat
Source: PeerJ. 2020 Sep 2;8:e9634. doi: 10.7717/peerj.9634 (PMC7474524; doi:10.7717/peerj.9634)
Supplement: Supplemental Information 4 — Key to museum abbreviations as in Table S1. [file peerj-08-9634-s004.docx]

| Order | Family | Taxon | Spec.# | Common name | Abbr. | Loc. | PH | DH | PU | PR | PF | DF | PT |
| --- | --- | --- | --- | --- | --- | --- | --- | --- | --- | --- | --- | --- | --- |
|  |  |  |  |  |  |  |  |  |  |  |  |  |  |
| Rodentia | Caviidae | *Cavia porcellus* | MCZ 6945 | guinea pig | Cavia | T |  |  | X |  | X |  |  |
| (Caviomorpha) |  | *Cavia porcellus* | UCMP 123701 | guinea pig | Cavia | T | X | X |  | X |  | X | X |
|  |  | *Kerodon rupestris* | FMNH 20236 | rock cavy | Kerod | T | X | X | X |  | X | X | X |
|  | Chinchillidae | *Chinchilla lanigera* | MCZ 47109 | long-tailed chinchilla | Chin | T | X | X | X |  | X | X | X |
|  |  | *Chinchilla lanigera* | UCMP 123716 | long-tailed chinchilla | Chin | T |  |  |  | X |  |  |  |
|  | Cuniculidae | *Cuniculus paca* | MCZ 829 | lowland paca | Cunic | T |  |  |  | X |  |  | X |
|  | Dasyproctidae | *Dasyprocta punctata* | MCZ 5094 | Central American agouti | Dasypro | T | X | X | X |  | X | X |  |
|  | Echimyidae | *Echimys* sp. | UCMP 841 | spiny rat | Echimys | A |  |  |  | X |  |  | X |
|  |  | *Toromys grandis* | MCZ 32353 | giant tree rat | Torom | A | X | X | X |  | X | X |  |
|  | Erethizontidae | *Erethizion rufescens* | MCZ 36327 | stump-tailed porcupine | Ereth | A | X | X | X | X | X | X |  |
|  |  | *Coendou spinosus (=villosus)* | MCZ 6025 | Paraguayan hairy dwarf porcupine | Coendu | A | X | X | X | X | X | X |  |
|  |  |  |  |  |  |  |  |  |  |  |  |  |  |
| Rodentia | Cricetidae | *Microtus* sp. | UCMP 58294 | vole | Microtus | T | X | X | X | X | X | X |  |
| (Myomorpha) |  | *Neotoma cinerea* | UCMP 58812 | bushy-tailed woodrat | Neotc | S |  |  | X | X |  |  |  |
|  |  | *Neotoma albigula* | MCZ 61188 | white-throated woodrat | Neota | S | X | X | X |  | X | X |  |
|  |  | *Reithrodontomys megalotis* | UCMP 123876 | Western harvest mouse | Reithro | T |  |  | X |  |  | X |  |
|  |  | *Reithrodontomys megalotis* | MCZ 60359 | Western harvest mouse | Reithro | T | X | X |  |  | X |  |  |
|  | Geomyidae | *Thomomys bottae* | UCMP 138321 | Botta’s pocket gopher | Thomo | T |  | X | X | X |  |  | X |
|  |  | *Thomomys bottae* | MCZ 52884 | Botta’s pocket gopher | Thomo | T | X |  |  |  | X | X |  |
|  | Gliridae | *Glis glis* | UCMP 123745 | edible or fat dormouse | Glis | A | X | X |  | X |  | X | X |
|  |  |  |  |  |  |  |  |  |  |  |  |  |  |
| Rodentia | Sciuridae | *Callospermophilus lateralis* | MCZ 11696 | golden-mantled ground squirrel | Callo | S | X | X | X | X | X | X | X |
| (Sciuromorpha) |  | *Heliosciurus rufobrachium* | MCZ 35326 | red-legged ground squirrel | Helios | S | X | X | X | X | X | X | X |
|  |  | *Marmota monax* | MCZ 852 | marmot | Marmot | T | X | X | X | X | X | X |  |
|  |  | *Protoxerus stangeri* | MCZ 53052 | African giant ground squirrel | Protox | S | X | X | X | X | X | X | X |
|  |  | *Sciurus carolinensis* | MCZ 61900 | Eastern gray squirrel | Sciur | S | X |  | X |  | X | X |  |
|  |  | *Sciurus carolinensis* | MCZ 63329 | Eastern gray squirrel | Sciur | S | X | X |  | X | X | X | X |
|  |  | *Sciurus carolinensis* | MCZ 65944 | Eastern gray squirrel | Sciur | S | X | X |  |  |  |  |  |
|  |  | *Tamias striatus* | MCZ 62976 | Eastern chipmunk | Tamias | T | X | X | X | X | X | X | X |
|  |  | *Tamiasciurus hudsonicus* | MCZ 60839 | American red squirrel | Tamiasc | S | X | X | X |  | X | X |  |
