# Supplementary material for: Postcranial elements of small mammals as indicators of locomotion and habitat
Source: PeerJ. 2020 Sep 2;8:e9634. doi: 10.7717/peerj.9634 (PMC7474524; doi:10.7717/peerj.9634)
Supplement: Supplemental Information 5 — Key to museum abbreviations as in Table S1. [file peerj-08-9634-s005.docx]

| Order | Family | Taxon | Spec.# | Common name | Abbr. | Loc. | PH | DH | PU | PR | PF | DF | PT |
| --- | --- | --- | --- | --- | --- | --- | --- | --- | --- | --- | --- | --- | --- |
|  |  |  |  |  |  |  |  |  |  |  |  |  |  |
| Carnivora | Mephitidae | *Spilogale putorius* | AMNH 135961 | Eastern spotted skunk | Spilog | T | X | X | X | X | X | X | X |
| (Caniformia) | Mustelidae | *Martes pennanti* | MCZ 56169 | fisher | Martes | S | X | X | X | X | X | X | X |
|  |  | *Mustela nigripes* | MCZ 42737 | black-footed ferret | Must | T | X | X | X | X | X | X | X |
|  | Procyonidae | *Ailurus fulgens* | MCZ 64643 | red panda | Ailurus | A | X | X | X | X | X | X | X |
|  |  | *Bassaricyon alleni* | MCZ 37922 | Allen’s olingo | Bassac | A | X | X | X |  | X | X |  |
|  |  | *Bassariscus astutus* | MCZ 42162 | ring-tail cat | Bassas | S | X | X | X | X | X | X | X |
|  |  | *Nasua nasua* | MCZ 1000 | South American coati | Nasua | S | X | X | X | X | X | X | X |
|  |  | *Potos flavus* | MCZ 62043 | kinkajou | Potos | A | X | X | X | X | X | X | X |
|  |  | *Procyon lotor* | MCZ 61027 | raccoon | Procyon | S | X | X | X | X | X | X | X |
|  |  |  |  |  |  |  |  |  |  |  |  |  |  |
| Carnivora | Eupleridae | *Eupleres goudotii* | MCZ 45958 | Eastern falanouc | Eupleres | T | X | X | X | X | X | X | X |
| (Feliformia) |  | *Fossa fossana* | FMNH 85196 | fanaloka | Fossa | T | X | X | X | X | X | X | X |
|  |  | *Galidia elegans* | FMNH 619717 | ring-tailed vontsira | Galide | S | X | X | X | X | X | X | X |
|  |  | *Galidictis fasciata* | FMNH 178720 | broad-striped vontsira | Galidf | T | X | X | X | X | X | X | X |
|  |  | *Mungotictis decemlineata* | FMNH 176128 | narrow-striped mongoose | Mungod | S | X | X | X |  | X | X | X |
|  | Herpestidae | *Herpestes javanicus* | MCZ 63333 | small Indian mongoose | Herpes | T | X | X | X | X | X | X |  |
|  |  | *Mungos mungo* | AMNH 51601 | banded mongoose | Mungos | T | X | X | X | X | X | X |  |
|  |  | *Suricata suricatta* | MCZ 5115 | meercat | Suricat | T | X | X | X | X | X | X |  |
|  | Nandinidae | *Nandinia binotata* | AMNH 51461 | African palm civet | Nandi | A | X | X | X | X | X | X | X |
|  | Prionodontidae | *Prionodon linsang* | MCZ 36576 | banded linsang | Priono | S | X | X |  |  | X | X |  |
|  | Viverridae | *Genetta maculata* | AMNH 51518 | large-spotted genet | Genetta | S | X | X | X | X | X | X |  |
|  |  | *Paradoxurus hermaphroditus* | MCZ 1969 | Asian palm civet | Paradox | S | X | X | X | X | X | X | X |
|  |  | *Viverricula indica* | MCZ 45985 | small Indian civet | Viverr | T | X | X | X | X | X | X | X |
