# Supplementary material for: Postcranial elements of small mammals as indicators of locomotion and habitat
Source: PeerJ. 2020 Sep 2;8:e9634. doi: 10.7717/peerj.9634 (PMC7474524; doi:10.7717/peerj.9634)
Supplement: Supplemental Information 6 [file peerj-08-9634-s006.docx]

| **Proximal humerus** | |
| --- | --- |
| L1 | Posterolateral edge of greater tuberosity |
| L2 | Posterior contact of greater tuberosity and humeral head |
| L3 | Most posterior point of curvature of humeral head |
| L4 | Posterior contact of lesser tuberosity and humeral head |
| L5 | Posteromedial edge of lesser tuberosity |
| L6 | Anteromedial edge of lesser tuberosity |
| L7 | Anterior contact of lesser tuberosity and humeral head |
| L8 | Anterior contact of greater tuberosity and humeral head |
| L9 | Anterolateral edge of greater tuberosity |
| L10 | Point of maximum curvature of greater tuberosity edge |
|  |  |
| **Distal humerus** | |
| L1 | Proximolateral border of capitulum (capitular tail) |
| L2 | Highest (most proximal) point of the capitulum |
| L3 | Proximal border between capitulum and trochlea |
| L4 | Proximomedial border of the trochlea |
| L5 | Medial-most extension of the trochlea along the medial wall |
| L6 | Distomedial border of the trochlea |
| L7 | Distal border between capitulum and trochlea |
| L8 | Deepest (most distal) point of the capitulum |
| L9 | Proximodistal border of the capitulum. |
|  |  |
| **Proximal ulna** | |
| L1 | Base of anconeal process |
| L2 | Tip of anconeal process |
| L3 | Lowest point of depression of semilunar notch |
| L4 | Tip of coronoid process |
| L5 | Base of coronoid process |
| L6 | Point on posterior ulna shaft directly opposite point 5 |
| L7 | Point on posterior ulna shaft directly opposite point 3 |
| L8 | Point on posterior ulna shaft directly opposite point 1 |
|  |  |
| **Proximal radius** | |
| L1 | Medial-most extension |
| L2 | Posteromedial corner |
| L3 | Point of greatest indentation (or central point) of posterior edge |
| L4 | Posterolateral corner |
| L5 | Lateral-most extension |
| L6 | Anterolateral corner |
| L7 | Point of greatest indentation (or central point) of anterior edge |
| L8 | Anteromedial corner |
|  |  |
| **Proximal femur** | |
| L1 | Most medial point of curvature of femoral head at the level of the fovea |
| L2 | Most dorsal point of curvature of femoral head |
| L3 | Medial-most point of dorsal femoral neck (= dorsal base of femoral head) |
| L4 | Lateral-most point of dorsal femoral neck (= medial base of greater trochanter) |
| L5 | Tip of greater trochanter |
| L6 | Lateral-most point of femur beneath greater trochanter  (= third trochanter or equivalent location) |
| L7 | Medial side of femur a level of base of lesser trochanter |
| L8 | Tip of lesser trochanter |
| L9 | Base of ventral femoral neck |
| L10 | Most ventral point of curvature of femoral head |
|  |  |

| **Distal femur** | |
| --- | --- |
| L1 | Medial edge of medial condyle |
| L2 | Median edge of medial condyle |
| L3 | Median edge of lateral condyle |
| L4 | Lateral edge of lateral condyle |
| L5 | Deepest point of intercondylar notch |
| L6 | Highest (most anterior) point of medial patella ridge |
| L7 | Highest (most anterior) point of lateral patella ridge |
| L8 | Point of deepest depression of patella groove |
|  |  |
| **Proximal tibia** | |
| L1 | Deepest point of popliteal notch |
| L2 | Posteromedian border of lateral condyle |
| L3 | Posterolateral border of lateral condyle |
| L4 | Anterolateral border of lateral condyle |
| L5 | Lateral base of tibial tuberosity |
| L6 | Tip of tibial tuberosity |
| L7 | Medial base of tibial tuberosity |
| L8 | Anteromedial border of medial condyle |
| L9 | Posteromedial border of medial condyle |
| L10 | Posteromedian border of medial condyle |
|  |  |
