# Supplementary material for: Postcranial elements of small mammals as indicators of locomotion and habitat
Source: PeerJ. 2020 Sep 2;8:e9634. doi: 10.7717/peerj.9634 (PMC7474524; doi:10.7717/peerj.9634)
Supplement: Supplemental Information 7 — Log CS = log-transformed centroid size. Locomo. = locomotor categories. Df = degrees of freedom. SS = sum of squares. MS = mean squares. Rsq = R squared. F = F statistic. Z = Z score (effect size). Pr(>F) = p-value obtained from permutation. Values with significance levels <0.05 are in bold face type. [file peerj-08-9634-s007.docx]

| Proximal humerus |  | Df | SS | MS | Rsq | F | Z | Pr(>F) |
| --- | --- | --- | --- | --- | --- | --- | --- | --- |
|  | Log CS | 1 | 0.001086 | 0.001086 | 0.01608 | 1.2787 | 0.7645 | 0.227 |
|  | Locomo. | 2 | 0.004974 | 0.0024871 | 0.07363 | 2.9283 | 3.3882 | **0.001** |
|  | Interaction | 2 | 0.002042 | 0.0010208 | 0.03022 | 1.2019 | 0.703 | 0.241 |
|  | Residuals | 70 | 0.059452 | 0.0008493 | 0.88007 |  |  |  |
|  | Total | 75 | 0.067554 |  |  |  |  |  |
| Distal humerus |  | Df | SS | MS | Rsq | F | Z | Pr(>F) |
|  | Log CS | 1 | 0.000522 | 0.0005218 | 0.00939 | 0.7482 | -0.36359 | 0.645 |
|  | Locomo. | 2 | 0.00277 | 0.0013852 | 0.04984 | 1.9864 | 2.04932 | **0.015** |
|  | Interaction | 2 | 0.002087 | 0.0010436 | 0.03755 | 1.4965 | 1.32577 | 0.082 |
|  | Residuals | 72 | 0.050209 | 0.0006973 | 0.90323 |  |  |  |
|  | Total | 77 | 0.055588 |  |  |  |  |  |
| Proximal ulna |  | Df | SS | MS | Rsq | F | Z | Pr(>F) |
|  | Log CS | 1 | 0.03296 | 0.032962 | 0.03067 | 2.4147 | 1.76735 | **0.026** |
|  | Locomo. | 2 | 0.09527 | 0.047637 | 0.08864 | 3.4897 | 3.05297 | **0.001** |
|  | Interaction | 2 | 0.03194 | 0.01597 | 0.02972 | 1.1699 | 0.58022 | 0.279 |
|  | Residuals | 67 | 0.91461 | 0.013651 | 0.85097 |  |  |  |
|  | Total | 72 | 1.07479 |  |  |  |  |  |
| Proximal radius |  | Df | SS | MS | Rsq | F | Z | Pr(>F) |
|  | Log CS | 1 | 0.0007262 | 0.0007262 | 0.03154 | 1.8966 | 1.396 | 0.067 |
|  | Locomo. | 2 | 0.0011209 | 0.0005605 | 0.04869 | 1.4637 | 1.10334 | 0.126 |
|  | Interaction | 2 | 0.0008822 | 0.0004411 | 0.03832 | 1.1521 | 0.55407 | 0.305 |
|  | Residuals | 53 | 0.0202931 | 0.0003829 | 0.88145 |  |  |  |
|  | Total | 58 | 0.0230224 |  |  |  |  |  |
| Proximal femur |  | Df | SS | MS | Rsq | F | Z | Pr(>F) |
|  | Log CS | 1 | 0.09821 | 0.098206 | 0.0522 | 4.2068 | 2.6019 | **0.005** |
|  | Locomo. | 2 | 0.19916 | 0.09958 | 0.10585 | 4.2657 | 3.3959 | **0.001** |
|  | Interaction | 2 | 0.04342 | 0.02171 | 0.02308 | 0.93 | 0.0402 | 0.476 |
|  | Residuals | 66 | 1.54073 | 0.023344 | 0.81888 |  |  |  |
|  | Total | 71 | 1.88152 |  |  |  |  |  |
| Distal femur |  | Df | SS | MS | Rsq | F | Z | Pr(>F) |
|  | Log CS | 1 | 0.02015 | 0.020152 | 0.01781 | 1.5039 | 0.90287 | 0.189 |
|  | Locomo. | 2 | 0.13949 | 0.069746 | 0.12326 | 5.205 | 3.1054 | **0.002** |
|  | Interaction | 2 | 0.03407 | 0.017037 | 0.03011 | 1.2715 | 0.66208 | 0.265 |
|  | Residuals | 70 | 0.93799 | 0.0134 | 0.82883 |  |  |  |
|  | Total | 75 | 1.13171 |  |  |  |  |  |
| Proximal tibia |  | Df | SS | MS | Rsq | F | Z | Pr(>F) |
|  | Log CS | 1 | 0.0281 | 0.028102 | 0.02837 | 1.4034 | 0.93828 | 0.188 |
|  | Locomo. | 2 | 0.0588 | 0.029398 | 0.05936 | 1.4681 | 1.22511 | 0.106 |
|  | Interaction | 2 | 0.06258 | 0.031289 | 0.06318 | 1.5625 | 1.4807 | 0.064 |
|  | Residuals | 42 | 0.84103 | 0.020025 | 0.84909 |  |  |  |
|  | Total | 47 | 0.99051 |  |  |  |  |  |
